# EcoTILLING by sequencing reveals polymorphisms in genes encoding starch synthases that are associated with low glycemic response in rice

**DOI:** 10.1186/s12870-016-0968-0

**Published:** 2017-01-14

**Authors:** Ramadoss Bharathi Raja, Somanath Agasimani, Sarita Jaiswal, Venkatesan Thiruvengadam, Robin Sabariappan, Ravindra N. Chibbar, Sundaram Ganesh Ram

**Affiliations:** 1 Centre for Plant Breeding and Genetics, Tamil Nadu Agricultural University, Coimbatore, 641 003 Tamil Nadu India; 2 Department of Plant Sciences, University of Saskatchewan, Saskatoon, SK S7N 5A8 Canada

**Keywords:** EcoTILLING by sequencing, Allele mining, Glycemic response, Rice, Resistant starch, Starch biosynthesis

## Abstract

**Background:**

Glycemic response, a trait that is tedious to be assayed in cereal staples, has been identified as a factor correlated with alarmingly increasing prevalence of Type II diabetes. Reverse genetics based discovery of allelic variants associated with this nutritional trait gains significance as they can provide scope for genetic improvement of this factor which is otherwise difficult to target through routine screening methods.

**Results:**

Through EcoTILLING by sequencing in 512 rice accessions, we report the discovery of six deleterious variants in the genes with potential to increase Resistant Starch (RS) and reduce Hydrolysis Index (HI) of starch. By deconvolution of the variant harbouring EcoTILLING DNA pools, we discovered accessions with a minimum of one to a maximum of three deleterious allelic variants in the candidate genes.

**Conclusions:**

Through biochemical assays, we confirmed the potential role of the discovered alleles alone or in combinations in increasing RS the key factor for reduction in glycemic response.

**Electronic supplementary material:**

The online version of this article (doi:10.1186/s12870-016-0968-0) contains supplementary material, which is available to authorized users.

## Background

Rice is the most important cereal staple for more than half the world’s population. As a primary dietary source of carbohydrates, it plays an important role in meeting energy requirements and nutrient intake among the rice eating populations [1]. Cooked rice is readily digested because it contains higher proportions of digestible starch (DS) and a lower RS [2]. RS has been reported by many studies to play an inhibitory role in the interaction of α amylase a predominant starch metabolising enzyme in human gut, with the carbohydrates in many cereals including rice resulting in slow digestibility of starch [3]. RS in cereal grains is reported to be the functional equivalent of dietary fibre through many animal studies [4–7].

In the past, dietary carbohydrates have been derived from whole coarse grains of rice, which were loaded with sufficient dietary fibre. At present, they are replaced predominantly with milled white rice carbohydrates devoid of any dietary fibre [8–10]. Studies involving human subjects related to the assessment of the causative factors for high prevalence of type II diabetes in Asia had indicated the consumption of milled white rice as one of the major factor [11–13]. The uninhibited interaction of α amylase with the carbohydrates from milled white rice leading to rapid release of glucose in the blood stream was demonstrated as the mechanism for diabetes incidence in many animal studies [6, 14–17].

Increasing the RS levels in the endosperm of cereal staples including rice is envisaged as an essential target for quality improvement of their starch in the context of human health [18]. Characterisation studies of cereal starches with high RS had indicated two major biochemical components to be positively associated with this desirable fraction. Studies of Miller et al. [19], Leeman et al. [20] and Lehman and Robin [21] had provided conclusive evidences for positive correlation of amylose with RS enhancement. While other characterisation studies in cereals had demonstrated that increased proportion of short chains and decrease in intermediate and long chain amylopectin also play a vital role for increase in RS content [22].

In rice, it is surprising to note that many of the *indica* varieties in spite of their intermediate to high amylose content (AC) (20–30%) in their grains do not show much reduction in their starch digestibility and remains rapid in their glycemic response [23]. Findings of Chung et al. [24] based on their study of rice varieties with varied amylose contents clearly indicated that apart from AC the higher proportion of short chain amylopectin is also a critical factor for reduction in digestibility of starch. This warrants the need for exploration of rice varieties with high AC along with increased proportion of short chain amylopectin to reduce its glycemic response.

Natural allelic variants are more stable in their expression as compared to induced mutations, as they are generated and stabilised over their long course of evolution [25]. The classical example of isolation and use of path breaking natural gene variants is the discovery of dwarfing genes such as Dee-geo-woo-gen in rice and Norin 10 in wheat which led to the green revolution during 1960s [26]. Recently, the isolation of *sub-1* gene leading to the development of submergence tolerant rice varieties is also a demonstration of the discovery and use of natural allelic variants from germplasm [27]. As the natural variants occur in an extremely low frequency, the power of allele mining to discover them has to be enhanced by applying modern genomic tools. Genomics assisted allele mining approaches when applied in reverse genetic mode results in enhanced power of detection and provides scope for high throughput screening of large germplasm in a short time frame [28]. Isolation of natural sequence allelic variants in targeted candidate genes has been successfully demonstrated through EcoTILLING in many plants such as *Arabidopsis* [29], banana [30], *Populus* [31], field bean [32], mung bean [33], barley [34], potato [35], *Cucumis* spp [36], tomato [37], Sugar beet [38] and also in rice [39].

The conventional TILLING and EcoTILLING methods using *CELI* endonuclease based heteroduplex cleavage are less effective and labour intensive, hence very challenging in employing them in large mutant and germplasm DNA pools. To overcome the difficulties of conventional TILLING approach, Tsai et al. [40] demonstrated TILLING by high throughput sequencing in large mutant populations of rice and wheat. Recently, TILLING by sequencing was also been employed for the identification of allelic variants responsible for abiotic and biotic resistance in peanut [41].

In the present investigation, we employed EcoTILLING by sequencing of candidate genes for the discovery of potential nucleotide variations associated with low glycemic response in rice. Our candidate gene selection was based on the studies of Sestili et al. [42], Regina et al. [43] and Satoh et al. [44] in wheat, barley and rice mutants generated through gene silencing and knock out technologies. These studies reported many potential loss of function mutations in the genes coding for Starch Synthases (SS) and Starch Branching Enzymes (SBEs) associated with the enhancement of RS.

## Results

### Variant discovery through EcoTILLING by sequencing

**Fig. 1:**
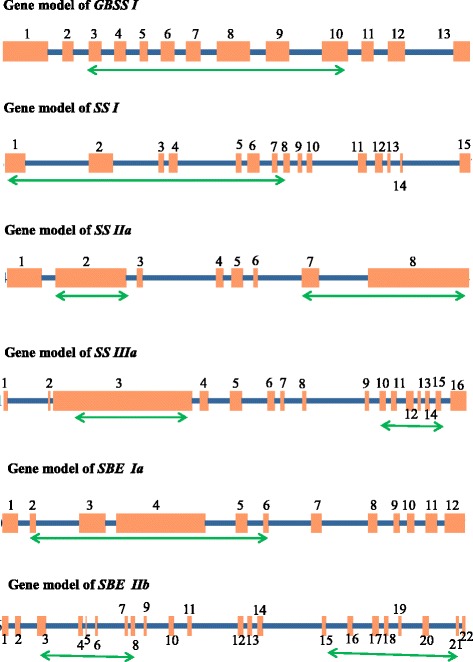
Gene models fragments discovered in the candidate genes for EcoTILLING. *Orange boxes* correspond to exons, lines to introns. (*Double arrow* showing region of EcoTILLING fragment

**Fig. 2:**
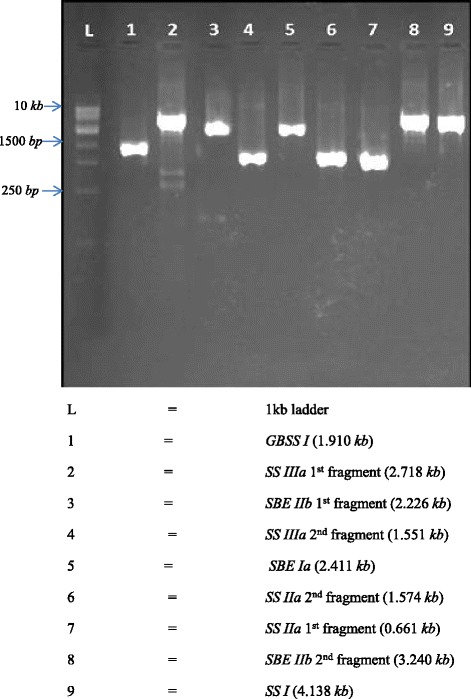
PCR amplification of nine EcoTILLING fragments covering six candidate genes

To identify the natural allelic variants in the starch biosynthesis genes of rice, we performed EcoTILLING by sequencing in 512 *indica* rice germplasm accessions representing landraces, breeding lines, cultivars and exotic collections (Additional file 1: Table S1). The identified EcoTILLING regions in all the six candidate genes with high probability to harbour variants as indicated by their high Position Specific Scoring Matrix (PSSM) difference were presented in Table 1. The position and the length of the EcoTILLING fragments of all six candidate genes were indicated in Fig. 1. EcoTILLING fragments were successfully amplified using targeted primers (Additional file 1: Table S2) through touch down PCR to minimize the off target amplifications as recommended by Don et al. [45] (Fig. 2). Various cycling conditions and master mix combinations were optimised for different candidate genes (Additional file 2: Table S4, Additional file 3: Table S5). The amplified PCR products were cleaned up and pooled to produce 16 libraries. The libraries were individually bar-coded, pooled and sequenced to assess the variants.

**Table 1:** Selected candidate genes with their functions and details of EcoTILLING fragments discovered

S. No	Gene	NCBI Genbank		Gene size (kb)	Gene area of highest PSSM Score difference	PSSM Score difference	No of amplicons used to cover EcoTILLING fragments	No of exons covered	No of splice sites covered	Function	Consequence of disruption
		mRNA ID	Genomic ID								
1.	*GBSS I*	AB425323.1	NC_008399.2	3.479	1679 to 2977	2782	1	8	7	Amylose biosynthesis	Down or up regulation of amylose
2.	*SS I*	GQ150950.1	NC_008399.2	6.815	4550 to 5848	1708	1	8	7	Amylopectin synthesis	Branching pattern of amylopectin is likely to be changed, no effect on amylose
3.	*SS IIa*	GQ150968.1	NC_008399.2	4.419	3037 to 4335	3346	2	3	1	Amylopectin synthesis	Branching pattern of amylopectin is likely to be changed, Increase in amylose
4.	*SS IIIa*	GQ151010.1	AK061604	11.083	8887 to10185	3203	2	7	5	Amylopectin synthesis	Branching pattern of amylopectin is likely to be changed, moderate increase in AC, no effect on RS
5.	*SBE Ia*	GQ150912.1	NC_008399.2	4.745	923 to 2221	2384	1	5	4	Amylopectin synthesis	Preferentially branches amylose type polyglucans. And it has capacity for branching less branched α-glucans. Also modifies amylopectin architecture and grain morphology.
6.	*SBE IIb*	D16201.1	NC_008395.2	10.899	3517 to 4815	1711	2	13	11	Amylopectin synthesis	Increased amylose and RS concentration and alter amylopectin architecture such as degree of branching and branching pattern

The average reads generated by Ion Proton sequencing from 16 super pooled DNA libraries varied from 2.20 to 6.96 million, with average read length varying from 81 to 98 bp. (Additional file 4: Table S6). The average depth of coverage per accession was 264.09, which had surpassed the suggested minimum reads of 10 X [40] per base indicating the variants discovered in this investigation possess very high confidence limits.

From 20.4 kb of EcoTILLING regions spanning in six candidate genes, 72 (60 SNPs and 12 single base Indels) natural variants were discovered (Additional file 5: Table S7). Out of the 60 SNPs, transitions accounted for 13 numbers each of T → C and G → A, followed by eight numbers of A → G, and six numbers of C → T. Seven transversions each of T → G and C → A followed by three numbers of T → A, two of G → C and one of A → C were observed. All the 12 single base Indels discovered were deletions.

### Prediction of deleterious variants

**Fig. 3:**
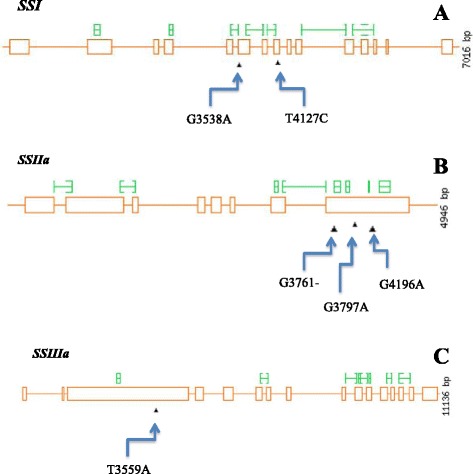
Overview of missense variants discovered in this study. The exon regions of the genes are represented by *yellow* boxes, while *yellow* lines shows intron region of the gene. **a** Position and nucleotide change of functional variants discovered in *SSI*. **b** Position and nucleotide change of functional variants discovered in *SSIIa*. **c** Position and nucleotide change of functional variants discovered in *SSIIIa*

The positional analysis of the nucleotide variants indicated that 23.6% of them were in the exons and 76.4% were present in introns. Further functional analysis of the exon mutations indicated that 64.8% were silent and 35.2% were deleterious variants. The predicted deleterious variants along with their deconvolved accessions were furnished in Table 2 and Fig. 3. Four sequence variants observed in the *GBSSI* gene were regarded as null mutants as they were synonymous for amino acid changes. Seven variants of *SSI* gene were exon residing SNPs, which included two missense and five silent variants. The amino acid substitutions predicted *viz*., Glycine → Serine at 319th residue in the accessions Os-578 and Os-631 and Tyrosine → Histidine at 420th residue in the accessions Os-076, Os-468 and Os-678 resulting from single base substitutions G3538A and T4127C, respectively were found to be deleterious with SIFT scores of 0.00. Two out of the four SNPs discovered in *SSIIa* gene (G3797A and G4196A) were missense variants and both were predicted as deleterious with SIFT score of 0.00 and they resulted in amino acid changes Glycine → Serine at 604th residue in accessions Os-211 and Os-468 and Valine → Methionine at 737th residue in the accessions Os-365 and Os-495, respectively. Furthermore, a single base deletion (G3761-) was also found to be deleterious in the accession Os-351 which resulted in frame shift. In the gene *SSIIIa,* a single nucleotide variant (T3559A) borne by the accessions Os-468, Os- 495 and Os-578 resulted in the alteration of amino acid Valine to Glutamic acid at 843rd position of the protein was also deleterious. Even though there were eight sequence variants observed in *SBEIa* and *SBEIIb*, none of them were predicted to be deleterious to protein function by SIFT analysis.

**Table 2:** Deleterious and synonymous variants discovered in the candidate genes through EcoTILLING by sequencing

S. No	Candidate gene	Nucleotide change^a^	Effect on protein sequence^$^	Restriction sites		SIFT score	Allele designation	Germplasm accessions deconvolved through Sanger sequencing
				Gained in variant	Lost from reference			
Deleterious variants								
1.	*SSI*	G3538A	G319S	*BsrI, TatI*	*Cfr10I, HpaII*	0.00	*SSI-1*	Os-578, Os-631
2.	*SSI*	T4127C	Y420H	*-*	*-*	0.00	*SSI-1*	Os-076, Os-468, Os-678
3.	*SSIIa*	G3761-	G592 *fs*	-	-	Frame shift	*SSIIa-1*	Os-351
4.	*SSIIa*	G3797A	G604S	*GsuI*	*HpaII*	0.00	*SSIIa-2*	Os-211, Os-468
5.	*SSIIa*	G4196A	V737M	*NlaIII*	*Tsp4CI*	0.00	*SSIIa-3*	Os-363,Os-495
6.	*SSIIIa*	T3559A	V843E		*TspDTI*	0.00	*SSIIIa-1*	Os-468, Os-495,Os-578
Positive control								
7.	*SSI*	G3538A	G319S	*BsrI, TatI*	*Cfr10I, HpaII*	0.00	*SSI-1*	RSM 311
8.	*SSIIa*	G3797A	G604S	*GsuI*	*HpaII*	0.00	*SSIIa-2*	RSM 271
9.	*SSIIa*	C978G	D283E	-	-	0.00	*SSIIa-4*	RSM 311
10.	*SSIIIa*	C1615T	A195V	-	-	0.00	*SSIIIa-2*	RSM 311, RSM 271
11.	*SSIIIa*	T3559A	V843E	-	*TspDTI*	0.00	*SSIIIa-1*	RSM 311, RSM 271
12.	*SSIIIa*	C10761T	T1755I	-	-	0.00	*SSIIIa-3*	RSM 311
Synonymous variants								
1.	*GBSSI*	T1804C	P362=	*BseYI*	*MaeI*	1.00	No effect	Os-599
2.	*SSI*	T1449C	L181=	*ApaI, BseSI,*	*-*	1.00	No effect	Os-441
3.	*SSI*	T2252C	P229=	*CauII, HpaII*	*EcoRII*	1.00	No effect	Os-008
4.	*SSI*	C2288A	G241=	*-*	*-*	1.00	No effect	Os-048
5.	*SSI*	T2432A	I260=	*-*	*Hpy178III,TspEI*	1.00	No effect	Os-107
6.	*SSI*	G3937A	E388=	*TspDTI*	*AluI, BseSI, CviJI,*	1.00	No effect	Os-425
7.	*SSIIa*	T516C	D129=	*FnuDII, HgaI, MaeIII, Tsp45I*	*SfaNI*	1.00	No effect	Os-178
8.	*SSIIa*	T3901G	G638=	-	-	1.00	No effect	Os-433
9.	*SSIIIa*	T2276C	H415=	-	*AvaIII, NlaIII*	1.00	No effect	Os-058
10.	*SSIIIa*	C3135A	R702=		-	1.00	No effect	Os-100
11.	*SBEIa*	C1569A	G304=	*BciVI*	*CviJI*	1.00	No effect	Os-717

^a^The letter on the left side of the numeral indicates the nucleotide of the wild type; letter on the right side of the numeral indicates the altered variant nucleotide; numeral indicates the base pair position of the nucleotide change with respect to the gene sequence; “-” indicates single base pair deletion

^$^“=” indicates a synonymous; the numeral indicates the residual number of the amino acid based on the gene model; letter on the left of the numeral indicates wild type amino acid; letter on the right side of the numeral indicates the altered amino acid; “fs” indicates the frameshift in the amino acid sequence

All the deleterious variants in this investigation were predicted with SIFT (Sorting Intolerant from Tolerant), a powerful bioinformatic pipeline that predicts whether an amino acid substitution affects protein function or not. It works with an algorithm which accounts for the tolerance of amino acid substitutions with relation to their physical properties. The predicted SIFT score ranges from 0 to 1. The amino acid substitution is predicted to be damaging if the score is < 0.05, and tolerated if the score is > 0.05.

### Biochemical characterisation

Grains from germplasm accessions carrying deleterious variants along with two positive control mutants (RSM 271 and RSM 311) and negative control rice cultivar Pooja were subjected to biochemical analysis. Results pertaining to the parameters related to starch digestibility are presented in Table 3. The cultivar Pooja, with no variants in all the *SS* genes, recorded lowest RS content of 2.5% and highest HI of 58.2%. The RS content of accessions carrying SNP variants in a single *SS* gene (Os-076, Os-211, Os-351, Os-631, Os-363, and Os-678) varied from 4.1 to 6.1% and was found to be moderately high in their HI (40.8 to 47.7%). Accessions with variants in two *SS* genes (Os-495, Os-578 and RSM 271) registered higher values of RS (6.8 to 7.4%) and relatively lower HI (42.3 to 46.5%). The accessions with SNP variants in all the three SS genes (Os-468 and RSM 311) were found to possess highest RS contents (7.5 to 7.6%) and registered very low HI values (36.3 to 37.8%).

**Table 3:** Biochemical characterization of germplasm accessions with functional variants discovered through EcoTILLING by sequencing

S. No.	Accessions	Allelic combinations				Mean ± SE			
						Total starch (%)	AC (%)	RS (%)	HI (%)
		GBSSI	SSI	SS IIa	SS IIIa				
1.	Os-076	-	2	-	-	82.6 ± 0.3^a^	26.0 ± 0.4^b^	5.4 ± 0.3^bc^	40.8 ± 0.5^ab^
2.	Os-468	-	2	2	1	70.7 ± 0.5^h^	22.8 ± 0.4^f^	7.6 ± 0.4^a^	37.8 ± 0.6^b^
3.	Os-578	-	1	-	1	79.3 ± 0.4^de^	24.5 ± 0.5^c^	6.8 ± 0.4^a^	42.3 ± 0.5^ab^
4.	Os-631	-	1	-	-	74.5 ± 0.5^fg^	25.8 ± 0.4^b^	6.1 ± 0.2^b^	45.6 ± 1.4^ab^
5.	Os-678	-	2	-	-	75.2 ± 0.3^f^	27.2 ± 0.1^a^	5.3 ± 0.2^bc^	43.9 ± 0.6^ab^
6.	Os-351	-	-	1	-	73.5 ± 0.5^g^	23.5 ± 0.3^ef^	4.5 ± 0.3^cd^	47.7 ± 0.4^ab^
7.	Os-211	-	-	2	-	82.1 ± 0.5^ab^	23.5 ± 0.3^ef^	6.0 ± 0.5^b^	47.1 ± 0.6^ab^
8.	Os-363	-	-	3	-	80.9 ± 0.5^bc^	24.0 ± 0.2^cde^	4.1 ± 0.2^d^	47.3 ± 0.7^ab^
9.	Os-495	-	-	3	1	82.0 ± 0.4^ab^	23.7 ± 0.2^cdef^	6.9 ± 0.5^b^	42.5 ± 0.4^ab^
10.	RSM 271	-	-	4	1, 2	82.0 ± 0.4^ab^	23.3 ± 0.2^def^	7.4 ± 0.4^a^	46.5 ± 0.8^ab^
11.	RSM 311	-	1	5	1, 2, 3	80.0 ± 0.2^cd^	24.2 ± 0.2^cd^	7.5 ± 0.3^a^	36.3 ± 0.5^b^
12.	Pooja	-	-	-	-	78.3 ± 0.5^e^	23.2 ± 0.3^ef^	2.5 ± 0.3^e^	58.2 ± 0.3^a^

Different superscripts in the same column denote a statistically significant difference (*p* ≤ 0.05) for each accession

## Discussion

In this investigation, we attempt to unravel genetic factors responsible for slow digestibility of rice starch in order to utilise them in breeding this popular cereal for health benefits. Recently the reverse genetic approach, TILLING when performed with high throughput sequencing was very effective for detection of mutations in large rice and wheat mutant populations [40]. Eco-TILLING, also a reverse genetic method derived from the principles of TILLING is very useful for high throughput discovery of rare alleles in naturally evolved populations [46]. In this study, we employed EcoTILLING by sequencing for the first time in rice germplasm to discover rare alleles associated with slow starch digestibility.

In this investigation, we had discovered 72 natural variants representing 60 SNPs and 12 single base indels by exploring 20.4 kb of target gene sequences in 512 germplasm accessions. Among the candidate gene targets, we observed remarkably higher number of sequence variants (64) in the genes coding for starch synthases than that of starch branching enzymes (8). Similar trend in variant frequencies was reported by Kharabian-Masouleh et al. [47] wherein 286 variants in starch synthases and only 94 variants in starch branching enzymes were discovered in 233 rice breeding lines. High frequency of natural variants observed in starch synthases is postulated to the ability to compliment the loss of function of mutant forms by a wild type allele and *vice versa.* In contrary, the genes coding for starch branching enzymes possess non-redundant function hence lack the potential for complementation was demonstrated in *Arabidopsis* [48] and wheat [49].

Enhanced expression of short chain amylopectin was demonstrated to be associated with low glycemic response in many cereals [22]. An earlier study in rice revealed that a knock out mutant of *SSI* gene was observed to produce altered amylopectin composition in rice endosperm with a tendency for enhanced short chains without affecting the grain morphology and test weight [50]. Two natural *SSI* allelic missense variants isolated for the first time in this study, G3538A substitution with a Glycine → Serine alteration at 319th amino acid residue in the gemplasm accessions Os-578 and Os-631 and T4127C substitution with Tyrosine → Histidine at 420th residue in three accessions Os-076, Os-468 and Os-678 are expected to carry potential for altered short chain amylopectin composition. These natural allelic variants of *SSI* gene could be deployed for development of non-transgenic rice cultivars with lower glycemic index (GI).

In a comparative study between *indica* and *japonica* cultivars, Nakamura et al. [51] found that all the *japonica* accessions carried a serine residue instead of a glycine residue found in *indica* types at the 604th amino acid position resulting from a G3797A substitution in *SSIIa* gene. Upon characterization for their length of amylopectin, they found an increased proportion of short chain of DP 6–12 and decreased longer amylopectin chains with DP13-24 in all the *japonica* cultivars carrying this variant. The same G3797A substitution was discovered in the *indica* accessions of Os-211 and Os-468 for the first time in this study. This allele could also be deployed in *indica* rice breeding programmes for reducing GI in rice. Furthermore, the potential missense single base deletion variant (G3761-) resulting in a frame shift leading to loss of glycine residue at 592nd amino acid position could also be a potential allele for altering the glycemic response in rice.

The gene expression pattern analysis in many studies using *japonica* rice suggest that *SSIIIa* plays an important role during the starch filling phase of the developing endosperm by its contribution towards amylopectin synthesis [52–54]. It has been reported that the deleterious mutations in this gene can cause inefficiency in grain filling which results in loosely packed starch with high chalkiness [55]. In contrary, Fujita et al. [56] characterized two mutants of *SSIIIa* in *japonica* background through protein quantification studies. They found that the reduced activity of *SSIIIa* in the mutant endosperm was accompanied with a compensatory enhancement of *GBSSI* and *SSI* activities in both the mutants. In these mutants, they also reported a significant increase in the molar ratio of short chain amylopectin in comparison to their longer counter parts. In the accessions Os-468, Os-495 and Os-578, we had discovered a missense variant (T3559A) which resulted in the alteration of amino acid Valine to Glutamic acid at the 843rd position of the protein. These accessions were characterized to be free from chalkiness (data not shown). Lack of chalkiness in these accessions could be postulated to the compensatory mechanism of *GBSSI* and *SSI* which are reported to exhibit multi-fold expression in *indica* varieties leading to no or less yield penalty. Such a compensatory mechanism is also evident in the control mutants RSM 271 and RSM 311 with normal grain size and morphology without chalkiness in spite of being carriers of three and four deleterious variants in *SSIIIa* gene, respectively.

The grains from 12 germplasm accessions carrying deleterious variants were subjected to biochemical analysis for determination of RS content and digestibility of starch through in vitro enzymatic studies (Table 3). Amylose content, an important parameter positively associated with RS expression, varied from intermediate to high (22.8 to 27.2%). Absence of low and waxy amylose types can be attributed to the lesser or no deleterious variants in the *GBSS I* gene which is commonly observed in *indica* rice varieties. As GBSS I is the only gene postulated to govern amylose synthesis in rice [57] hence complementation for loss of function mutations is remote unlike in the case of other starch synthases (*SSI, SSII* and *SSIIIa*) governing amylopectin synthesis.

Test accessions in this study revealed considerable variation for RS (4.1 to 7.6%) and HI (37.8 to 47.7%) in spite of the lesser variation in AC. In contrary to many investigations in germplasm of cereals [58–60] which had indicated positive correlation of AC and RS, their association in this study was negative (*r* = -0.316). The reason may be that the previous studies had representative accessions in all AC classes including low amylose and waxy types.

It is interesting to note that amylose independent variation observed in the RS and HI among the intermediate and high AC types was found to be dependent on the number of variants harboured in each of the *SS* coding genes and also on number of genes that carry the variants. For example, in the control cultivar Pooja which do not harbour any variant in the *SS* coding genes recorded lowest RS content (2.5%) and highest HI (58.2%). The six accessions (Os-076, Os-211, Os-351, Os-363, Os-631 and Os-678,) carrying variants in a single gene expressed moderately higher values of RS (4.1 to 6.1%) and HI (43.9 to 47.7%), whereas the accessions (Os-495, Os-578 and RSM 271) with variants in two genes expressed high values of RS (6.0 to 6.8%) and relatively lower HI (42.3 to 42.5%). The accessions (Os-468 and RSM 311) with variants in all the three *SS* coding genes were found to possess very high RS value (7.6%) and very low in HI (37.8%). The hydrolysis index (HI) is an in vitro biochemical determinant that estimates the rate of starch digestion of starchy food stuffs [61]. Various authors have suggested in vitro starch hydrolysis methods can be useful for predicting in vivo glycemic response of starchy staples [62, 63].

An earlier study in rice had indicated that each SS coding gene plays a partially overlapping role in the synthesis of amylopectin fraction of starch. Zhang et al. [64] by repression of genes through RNAi established that *SSIIa* and *SSIIIa* interact with each other during starch synthesis leading to accumulation of amylopectin with variable molecular forms. In this investigation, we have isolated, to the best of our knowledge, for the first time a genotype Os-468 carrying mutations in all three SS coding genes *viz*., *SSI*, *SSIIa* and *SSIIIa* which also exhibited very high levels of RS (7.6%) and extremely low HI (37.8%) with a possible predominance of short chain amylopectin. This has to be proven by determination of the degree of polymerization (DP) of amylopectin of this elite germplasm line. The DP of amylopectin is a numerical indicator of chain length in terms of the number of constitutive monomeric glucose molecules. It determines many physico-chemical properties of grain starch which includes retrogradation behaviour, pasting and swelling properties, gelatinization temperature along with enzymatic digestibility [65–67]. Many studies had indicated that the fine structure of amylopectin can alter the digestibility rate of starch in rice. Yang et al. [68] in their study with rice mutants high in RS was found to exhibit an increased proportion of short chain amylopectin as compared to the proportion of long chains. Shu et al. [22] based on their study with six rice mutants with altered fine structure of amylopectin also established the similar relationship between RS content and increased proportion of short chain amylopectin with DP ≤ 12. Critical analysis of the structural chemistry of amylopectin in the genotype Os-468 will also provide concrete evidence for the postulated relationship between amylopectin fine structure with RS and starch digestibility.

## Conclusion

We conclude that EcoTILLING by sequencing is a robust tool to survey allelic variants in target genes across large germplasm panels in rice. Our discovery of accessions with multiple missense variants in genes encoding starch synthases has the potential to reduce the glycemic response of rice starch.

## Methods

### Plant materials

Seeds of 837 *Oryza sativa* germplasm accessions from 5 different continents (Asia, Africa, North America, South America and Australia) representing18 countries were obtained from two different sources *viz*., Paddy Breeding Station, Tamil Nadu Agricultural University (TNAU), Coimbatore, Tamil Nadu, India and Ramiah Gene Bank, Department of Plant Genetic Resources, TNAU, Coimbatore, India. Two high RS expressing mutants *viz.,* RSM 271 and RSM 311 isolated recently at our laboratory through gamma irradiation were included as positive controls*. A* rice cultivar Pooja with very low RS (unpublished) was included as a negative control for comparison. These accessions were raised in a single row trial. Based on the observations on flowering, seed set and plant morphology (data not shown), a total of 547 accessions were found to be photo insensitive and suitable for further multiplication. Out of 547 accessions, we randomly selected 512 accessions belonging to *indica* type for EcoTILLING by sequencing (Additional file 1: Table S1).

### DNA extraction and normalization

Total genomic DNA from chosen 512 accessions was extracted from the leaf tissues using DNeasy 96 Plant kit (Qiagen, Valencia, CA, USA) following the manufacturer’s protocol. The DNA concentration was measured with Tecan Infinite M200 pro multimode reader (Tecan, Switzerland) using a nano quant plate. After assessment of the concentration, DNA samples were normalized by dispensing different volumes of water in DNA samples using a Tecan Freedom Evo75 robotic liquid handling system (Tecan, Switzerland).

### Pooling and super pooling of genomic DNA

Bidimensional pooling strategy of Tsai et al. [40] was adopted with slight modifications. We combined equivalent amount of concentration normalized DNA from eight germplasm accessions to make one 64 well pool plate in a symmetrical 8 × 8 well format instead of the regular 8 × 12 (96 well) microplate format. Genomic DNAs were further pooled by collapsing rows (8 wells × 8 individuals = 64 individuals) and column (8 wells × 8 individuals = 64 individuals) of this plate which resulted in 16 template super pools.

### Selection of candidate genes and their sequences

Through literature search, we identified the putative candidate genes associated with RS expression in rice [54, 69–72]. The list of chosen genes with their putative functional effects on RS was presented in Table 1. The nucleotide sequences of gDNA and full length cDNAs of candidate genes were retrieved from the NCBI Genbank. Sequences of these genes were utilized for building up gene models and designing primers.

### Discovery of EcoTILLING fragments, designing primers and PCR amplification

The EcoTILLING gene regions with maximum probability for missense variants were fixed using CODDLE bioinformatics pipeline (http://blocks.fhcrc.org/proweb/). The primers for PCR amplification of EcoTILLING fragments were designed with PRIMER 3 software (Additional file 6: Table S2).

### PCR for amplification of EcoTILLING fragments

High fidelity LongAmp® Taq DNA polymerase (Cat#M0534) obtained from New England Biolabs (NEB), Ipswich, UK was used for PCR amplification in a 50 μl reaction. PCRs were performed using 10 μl of 5x longAMPTaq Reaction buffer, 1.5 μl of dNTPs (10 mM), 2 μl of each forward and reverse primer (10 μM), 2 μl of DMSO, 5 μl of pooled DNA (50 ng/μl), 2 μl of LongAmp® Taq polymerase (5 unit) and 25.5 μl sterile water (Additional file 7: Table S3). Touch down PCR cycling was performed with a 30 s 95 °C denaturing step followed by 10 touchdown cycles at 94 °C for 20 s, 62 °C for 1 min (decrement at 0.6 °C cycle^-1^), and 65 °C for 1 min. Thirty more cycles were followed at 94 °C for 30 s, 57 °C for 1 min, 65 °C for 1 min with 10 min final extension at 65 °C. Reactions were held at 10 °C until retrieved (Additional file 2: Table S4, Additional file 3: Table S5).

### Equimolar pooling of PCR products and sequencing of libraries

The concentration of PCR products were quantified using the Qubit dsDNA BR assay system (Invitrogen, Carlsbad, CA) to eliminate over-estimation resulting from free nucleotides in the PCR products. The amplified products of the EcoTILLING fragments were normalized and equimolarly pooled gene wise maintaining the super pool identity.

Sequencing library preparation was carried out using the Ion Xpress™ Fragment Library Kit, with 100 ng of super pooled DNA. Adapter ligation, size selection, nick repair and amplification were performed as per manufacturer’s instructions ((Ion Xpress™ Fragment Library Kit - Part Number 4469142Rev.B). Size selection was executed using the Lab Chip XT (Caliper Life Sciences, USA) and the Lab Chip XT DNA 750 Assay Kit (Caliper Life Sciences, USA), with collection between 175 bp and 220 bp. The Agilent 2100 Bioanalyzer (Agilent Technologies, USA) and the manufacture recommended high sensitivity DNA kit (Agilent Technologies, USA) were used to determine quality and concentration of the libraries. Emulsion PCR and enrichment steps were carried out using the Ion Xpress™ Template Kit adopting its associated protocol (Part Number 44 69004 Rev. B). Individual libraries were barcoded by using Ion Xpress™ Barcode Adapters Kit. Sequencing was carried out using Ion Proton™ with 10 GB data output by using Ion 316™ Chip. The Ion Sequencing Kit v2.0 was used for sequencing reactions of all 16 libraries as per manufacturer’s instructions.

### SNP calling and mutation discovery

After the sequencing of libraries, filtering, trimming and aligning of sequence information were carried out by using Torrent Suite 1.5 with their reference sequences. After the alignment, Variant Caller was used for filtering the SNPs from the aligned sequence contigs in comparison with their corresponding reference sequences. The parameters such as min-max distance, mismatch cost, length fraction and similarity were selected in order to minimize reads alignment ambiguities as well to detect rare SNPs. The minimum variant frequency and minimum coverage were set 0.5 and 20, respectively which gives variations on or above 0.5% from the pools which were considered as SNPs. The candidate gene sequences of Pooja (a line with lowest RS content of 2.5%) were used as reference for variant calling.

### Functional analysis of SNP variants

Discovered sequence variants were analysed by the PARSESNP program (http://blocks.fhcrc.org/proweb/) which provides information on the location along with the details about amino acid changes. The severity of mutations was analysed by SIFT (Sorting Intolerant from Tolerant) (http://sift.jcvi.org/) with default parameters [73]. Amino acids with substitutions probabilities <0.05 are predicted to affect protein function.

### Deconvolution fromEcoTILLING pools

To identify the individual germplasm accessions carrying natural allelic variants from the prospective pools, individual genomic DNA of the eight constituent accessions was subjected to PCR amplification with primers designed for short target (~600 bp) spanning the variant region for each target gene. Sanger sequencing was performed using BigDye® Terminator version 3.1 cycle sequencing kit (Applied Biosystems, USA) on an ABI3730L (96 well) sequencer (Applied Biosystems, USA) according to the manufacturer’s protocols. By comparing the gene sequences of the individual PCR amplicons after alignment with their reference sequence, the positive variant carrying accessions were identified for subsequent characterization.

### Biochemical characterization of variants

Biochemical traits measured were total starch content, amylose content (AC), resistant starch (RS) and hydrolysis index (HI). Total starch contents were determined on the basis of the AACC International (AACC Method 76–13.01) method. AC was determined through high performance size exclusion liquid chromatography as described by Demeke et al. [74]. The RS content was estimated on dry weight basis following Goni et al. [75] using the Megazyme RS assay kit (Cat#K-RSTAR; Megazyme International Ireland Ltd., Ireland). In vitro starch hydrolysis rate and HI were determined according to Goni et al. [61]. In vitro enzymatic hydrolysis with different time points (0, 30, 60, 120 and 240 min) were carried out to predict the rate of starch digestibility which is measured as HI by comparing the rate of digestibility of white bread. This is considered to be in vitro equivalent of GI estimate. All determinations were done in three biological replicates and two independent observations for each replicate.

### Statistical analysis

Duncan’s multiple range test (DMRT) was carried out using MINITAB 16 to distinguish the mean differences between the accessions.

## Additional files

Additional file 1: Table S1. Details of rice accessions selected for EcoTILLING by sequencing. (DOCX 46 kb)

Additional file 2: Table S4. PCR reaction conditions followed for the amplification of EcoTILLING fragments. (DOCX 14 kb)

Additional file 3: Table S5. PCR cycle condition and composition standardized for the amplification of different EcoTILLING fragments in candidate genes. (DOCX 14 kb)

Additional file 4: Table S6. Super pool wise sequencing statistics. (DOCX 15 kb)

Additional file 5: Table S7. Gene wise list of sequence variants discovered through EcoTILLING by sequencing. (DOC 107 kb)

Additional file 6: Table S2. Details of gene specific primers designed to amplify EcoTILLING fragments. (DOCX 14 kb)

Additional file 7: Table S3. PCR compositions followed for the amplification of EcoTILLING fragments. (DOCX 14 kb)

## Abbreviations

AC: Amylose content
DP: Degree of polymerization
DS: Digestible starch
GI: Glycemic index
HI: Hydrolysis index
PSSM: Position specific scoring matrix
RS: Resistant starch
SBE: Starch branching enzyme
SIFT: Sorting intolerant from torrent
SNP: Single nucleotide polymorphism
SS: Starch synthase
