# Supplementary material for: EcoTILLING by sequencing reveals polymorphisms in genes encoding starch synthases that are associated with low glycemic response in rice
Source: BMC Plant Biol. 2017 Jan 14;17:13. doi: 10.1186/s12870-016-0968-0 (PMC5423428; doi:10.1186/s12870-016-0968-0)
Supplement: Supplementary file 1 — Details of rice accessions selected for EcoTILLING by sequencing. (DOCX 46 kb) [file 12870_2016_968_MOESM1_ESM.docx]

**Table S1. Details of rice accessions selected for EcoTILLING by sequencing**

| **S.No** | **Accession number** | | **Species** | **Status** | **Origin** | |
| --- | --- | --- | --- | --- | --- | --- |
| 1 | **Os-001** | | *Oryza sativa* | Variety | TNAU, Coimbatore, TN, India | |
| 2 | **Os-002** | | *Oryza sativa* | Variety | IRRI, Philippines | |
| 3 | **Os-003** | | *Oryza sativa* | Variety | IRRI, Philippines | |
| 4 | **Os-005** | | *Oryza sativa* | Variety | TRRI, Aduthurai, TN, India | |
| 5 | **Os-006** | | *Oryza sativa* | Variety | TNAU, Coimbatore, TN, India | |
| 6 | **Os-007** | | *Oryza sativa* | Variety | TNAU, Coimbatore, TN, India | |
| 7 | **Os-008** | | *Oryza sativa* | Variety | ANGRAU,AP, India | |
| 8 | **Os-009** | | *Oryza sativa* | Variety | IRRI, Philippines | |
| 9 | **Os-010** | | *Oryza sativa* | Variety | TNAU, Coimbatore, TN, India | |
| 10 | **Os-011** | | *Oryza sativa* | Variety | TNAU, Coimbatore, TN, India | |
| 11 | **Os-012** | | *Oryza sativa* | Variety | Kerala, India | |
| 12 | **Os-014** | | *Oryza sativa* | Variety | KAU, Kerala. India | |
| 13 | **Os-015** | | *Oryza sativa* | Variety | TNAU, Coimbatore, TN, India | |
| 14 | **Os-016** | | *Oryza sativa* | Variety | IRRI, Philippines | |
| 15 | **Os-017** | | *Oryza sativa* | Variety | TRRI, Aduthurai, TN, India | |
| 16 | **Os-018** | | *Oryza sativa* | Variety | TRRI, Aduthurai, TN, India | |
| 17 | **Os-020** | | *Oryza sativa* | Variety | KAU, Kerala. India | |
| 18 | **Os-021** | | *Oryza sativa* | Variety | KAU, Kerala. India | |
| 19 | **Os-023** | | *Oryza sativa* | Variety | IRRI, Philippines | |
| 20 | **Os-026** | | *Oryza sativa* | Variety | TNAU, Coimbatore, TN, India | |
| 21 | **Os-027** | | *Oryza sativa* | Variety | ARS, Paramakudi, TNAU,India | |
| 22 | **Os-028** | | *Oryza sativa* | Variety | RRS, Tirur, TNAU, India | |
| 23 | **Os-029** | | *Oryza sativa* | Variety | TNAU, Coimbatore, TN, India | |
| 24 | **Os-030** | | *Oryza sativa* | Variety | TNAU, Coimbatore, TN, India | |
| 25 | **Os-031** | | *Oryza sativa* | Variety | IARI,New Delhi, India | |
| 26 | **Os-032** | | *Oryza sativa* | Variety | KAU, Kerala. India | |
| 27 | **Os-035** | | *Oryza sativa* | Variety | TNAU, Coimbatore, TN, India | |
| 28 | **Os-037** | | *Oryza sativa* | Variety | TRRI, Aduthurai, TN, India | |
| 29 | **Os-038** | | *Oryza sativa* | Variety | TNAU, Coimbatore, TN, India | |
| 30 | **Os-039** | | *Oryza sativa* | Variety | RRS, Tirur, TNAU, India | |
| 31 | **Os-040** | | *Oryza sativa* | Variety | TNAU, Coimbatore, TN, India | |
| 32 | **Os-041** | | *Oryza sativa* | Variety | TRRI, Aduthurai, TN, India | |
| 33 | **Os-042** | | *Oryza sativa* | Variety | CRRI, Cuttack | |
| 34 | **Os-043** | | *Oryza sativa* | Variety | RRS, Ambasamudram, TN, India | |
| 35 | **Os-044** | | *Oryza sativa* | Variety | RRS, Ambasamudram, TN, India | |
| 36 | **Os-045** | | *Oryza sativa* | Variety | West Bengal, India | |
| 37 | **Os-046** | | *Oryza sativa* | Variety | West Bengal, India | |
| 38 | **Os-048** | | *Oryza sativa* | Variety | Nagpur, Ankur Seed Pvt.Ltd. | |
| 39 | **Os-049** | | *Oryza sativa* | Variety | TNAU, Coimbatore, TN, India | |
| 40 | **Os-050** | | *Oryza sativa* | Variety | Nagina, UP, India | |
| 41 | **Os-051** | | *Oryza sativa* | Variety | TNAU, Coimbatore, TN, India | |
| 42 | **Os-052** | | *Oryza sativa* | Variety | TNAU, Coimbatore, TN, India | |
| 43 | **Os-053** | | *Oryza sativa* | Variety | TRRI, Aduthurai, TN, India | |
| 44 | **Os-055** | | *Oryza sativa* | Variety | India | |
| 45 | **Os-056** | | *Oryza sativa* | Variety | NA | |
| 46 | **Os-057** | | *Oryza sativa* | Variety | Tamil Nadu, India | |
| 47 | **Os-058** | | *Oryza sativa* | Variety | TNAU, Coimbatore, TN, India | |
| 48 | **Os-059** | | *Oryza sativa* | Variety | TNAU, Coimbatore, TN, India | |
| 49 | **Os-061** | | *Oryza sativa* | Variety | TNAU, Coimbatore, TN, India | |
| 50 | **Os-062** | | *Oryza sativa* | Variety | ARS, Paramakudi, TN | |
| 51 | **Os-063** | | *Oryza sativa* | Variety | TRRI, Aduthurai, TN, India | |
| 52 | **Os-064** | | *Oryza sativa* | Variety | NA | |
| 53 | **Os-068** | | *Oryza sativa* | Variety | TNAU, Coimbatore, TN, India | |
| 54 | **Os-069** | | *Oryza sativa* | Variety | CVRC, Jharkhand | |
| 55 | **Os-070** | | *Oryza sativa* | Variety | TNAU, Coimbatore, TN, India | |
| 56 | **Os-072** | | *Oryza sativa* | Variety | RRS, Ambasamudram, TN, India | |
| 57 | **Os-073** | | *Oryza sativa* | Variety | TNAU, Coimbatore, TN, India | |
| 58 | **Os-074** | | *Oryza sativa* | Variety | NA | |
| 59 | **Os-075** | | *Oryza sativa* | Variety | Eastern India | |
| 60 | **Os-076** | | *Oryza sativa* | Variety | NA | |
| 61 | **Os-077** | | *Oryza sativa* | Mutant line | TNAU, Coimbatore, TN, India | |
| 62 | **Os-078** | | *Oryza sativa* | Mutant line | TNAU, Coimbatore, TN, India | |
| 63 | **Os-079** | | *Oryza sativa* | Mutant line | TNAU, Coimbatore, TN, India | |
| 64 | **Os-080** | | *Oryza sativa* | Mutant line | TNAU, Coimbatore, TN, India | |
| 65 | **Os-081** | | *Oryza sativa* | Variety | TNAU, Coimbatore, TN, India | |
| 66 | **Os-082** | | *Oryza sativa* | Variety | NA | |
| 67 | **Os-083** | | *Oryza sativa* | Variety | NA | |
| 68 | **Os-085** | | *Oryza sativa* | Variety | NA | |
| 69 | **Os-086** | | *Oryza sativa* | Variety | NA | |
| 70 | **Os-088** | | *Oryza sativa* | Variety | Thailand | |
| 71 | **Os-089** | | *Oryza sativa* | Variety | NA | |
| 72 | **Os-091** | | *Oryza sativa* | Breeding line | NA | |
| 73 | **Os-092** | | *Oryza sativa* | Variety | NA | |
| 74 | **Os-093** | | *Oryza sativa* | Variety | Pakistan | |
| 75 | **Os-094** | | *Oryza sativa* | Variety | NA | |
| 76 | **Os-095** | | *Oryza sativa* | Variety | NA | |
| 77 | **Os-096** | | *Oryza sativa* | Variety | NA | |
| 78 | **Os-097** | | *Oryza sativa* | Variety | NA | |
| 79 | **Os-098** | | *Oryza sativa* | Variety | Maharastra | |
| 80 | **Os-099** | | *Oryza sativa* | Breeding line | Marasteru, ANGARC | |
| 81 | **Os-100** | | *Oryza sativa* | Variety | NA | |
| 82 | **Os-101** | | *Oryza sativa* | Variety | DRR, Hyderabad, India | |
| 83 | **Os-102** | | *Oryza sativa* | Variety | NA | |
| 84 | **Os-103** | | *Oryza sativa* | Variety | NA | |
| 85 | **Os-104** | | *Oryza sativa* | Variety | Rasi Seeds Pvt.Ltd, Attur | |
| 86 | **Os-105** | | *Oryza sativa* | Variety | Bengal, India | |
| 87 | **Os-106** | | *Oryza sativa* | Variety | NA | |
| 88 | **Os-107** | | *Oryza sativa* | Variety | MPKV, Rahuri | |
| 89 | **Os-108** | | *Oryza sativa* | Variety | Domesticated from China | |
| 90 | **Os-109** | | *Oryza sativa* | Breeding line | Local variety of Orissa | |
| 91 | **Os-110** | | *Oryza sativa* | Breeding line | NA | |
| 92 | **Os-111** | | *Oryza sativa* | Breeding line | Jagtial (ANGRAU), AP, India | |
| 93 | **Os-112** | | *Oryza sativa* | Variety | CSISA, Bangladesh, India | |
| 94 | **Os-113** | | *Oryza sativa* | Breeding line | RRS, Moncompu, KAU, Kerala, India | |
| 95 | **Os-114** | | *Oryza sativa* | Variety | NA | |
| 96 | **Os-115** | | *Oryza sativa* | Breeding line | NA | |
| 97 | **Os-118** | | *Oryza sativa* | Variety | Uganda, East Africa | |
| 98 | **Os-120** | | *Oryza sativa* | Breeding line | JNKVV, Jabalpur | |
| 99 | **Os-122** | | *Oryza sativa* | Land race | NA | |
| 100 | **Os-123** | | *Oryza sativa* | Breeding line | NA | |
| 101 | **Os-125** | | *Oryza sativa* | Variety | NA | |
| 102 | **Os-128** | | *Oryza sativa* | Breeding line | IRRI, Phillipines | |
| 103 | **Os-129** | | *Oryza sativa* | Land race | NA | |
| 104 | **Os-132** | | *Oryza sativa* | Variety | UAS, Bangalore | |
| 105 | **Os-134** | | *Oryza sativa* | Variety | NA | |
| 106 | **Os-136** | | *Oryza sativa* | Breeding | CSSRI, Karnal | |
| 107 | **Os-140** | | *Oryza sativa* | Breeding line | CSSRI, Karnal | |
| 108 | **Os-142** | | *Oryza sativa* | Breeding line | NA | |
| 109 | **Os-143** | | *Oryza sativa* | Breeding line | NA | |
| 110 | **Os-144** | | *Oryza sativa* | Breeding line | NA | |
| 111 | **Os-147** | | *Oryza sativa* | Breeding line | IRRI, Phillipines | |
| 112 | **Os-150** | | *Oryza sativa* | Breeding line | NA | |
| 113 | **Os-152** | | *Oryza sativa* | Breeding line | IRRI, Phillipines | |
| 114 | **Os-156** | | *Oryza sativa* | Breeding line | IRRI, Phillipines | |
| 115 | **Os-157** | | *Oryza sativa* | Breeding line | TNAU, Coimbatore, TN, India | |
| 116 | **Os-158** | | *Oryza sativa* | Variety | TNAU, Coimbatore, TN, India | |
| 117 | **Os-159** | | *Oryza sativa* | Variety | TNAU, Coimbatore, TN, India | |
| 118 | **Os-160** | | *Oryza sativa* | Variety | TNAU, Coimbatore, TN, India | |
| 119 | **Os-162** | | *Oryza sativa* | Variety | TNAU, Coimbatore, TN, India | |
| 120 | **Os-164** | | *Oryza sativa* | Variety | TNAU, Coimbatore, TN, India | |
| 121 | **Os-165** | | *Oryza sativa* | Variety | TNAU, Coimbatore, TN, India | |
| 122 | **Os-166** | | *Oryza sativa* | Variety | TNAU, Coimbatore, TN, India | |
| 123 | **Os-167** | | *Oryza sativa* | Variety | TNAU, Coimbatore, TN, India | |
| 124 | **Os-169** | | *Oryza sativa* | Variety | TNAU, Coimbatore, TN, India | |
| 125 | **Os-170** | | *Oryza sativa* | Variety | TNAU, Coimbatore, TN, India | |
| 126 | **Os-173** | | *Oryza sativa* | Variety | TNAU, Coimbatore, TN, India | |
| 127 | **Os-178** | | *Oryza sativa* | Variety | TNAU, Coimbatore, TN, India | |
| 128 | **Os-181** | | *Oryza sativa* | Variety | TNAU, Coimbatore, TN, India | |
| 129 | **OS-183** | | *Oryza sativa* | Variety | TNAU, Coimbatore, TN, India | |
| 130 | **Os-184** | | *Oryza sativa* | Variety | TNAU, Coimbatore, TN, India | |
| 131 | **Os-185** | | *Oryza sativa* | Variety | TNAU, Coimbatore, TN, India | |
| 132 | **Os-187** | | *Oryza sativa* | Variety | Southern Uninited States | |
| 133 | **Os-191** | | *Oryza sativa* | Variety | TRRI, TN, India | |
| 134 | **Os-193** | | *Oryza sativa* | Variety | Southern Uninited States | |
| 135 | **Os-197** | | *Oryza sativa* | Variety | Southern Uninited States | |
| 136 | **Os-200** | | *Oryza sativa* | Variety | TNAU, Coimbatore, TN, India | |
| 137 | **Os-202** | | *Oryza sativa* | Variety | TNAU, Coimbatore, TN, India | |
| 138 | **Os-204** | | *Oryza sativa* | Variety | TNAU, Coimbatore, TN, India | |
| 139 | **Os-206** | | *Oryza sativa* | Variety | TNAU, Coimbatore, TN, India | |
| 140 | **Os-209** | | *Oryza sativa* | Variety | TNAU, Coimbatore, TN, India | |
| 141 | **Os-211** | | *Oryza sativa* | Variety | TNAU, Coimbatore, TN, India | |
| 142 | **Os-213** | | *Oryza sativa* | Variety | TNAU, Coimbatore, TN, India | |
| 143 | **Os-216** | | *Oryza sativa* | Variety | TNAU, Coimbatore, TN, India | |
| 144 | **Os-217** | | *Oryza sativa* | Variety | Haryana, India | |
| 145 | **Os-218** | | *Oryza sativa* | Variety | TRRI, Aduthurai, TN, India | |
| 146 | **Os-220** | | *Oryza sativa* | Variety | TN, India | |
| 147 | **OS-223** | | *Oryza sativa* | Variety | NA | |
| 148 | **Os-225** | | *Oryza sativa* | Breeding line | NA | |
| 149 | **Os-226** | | *Oryza sativa* | Variety | AC & RI, Madurai, TN, India | |
| 150 | **Os-227** | | *Oryza sativa* | Variety | NA | |
| 151 | **Os-229** | | *Oryza sativa* | Variety | NA | |
| 152 | **Os-331** | | *Oryza sativa* | Breeding line | NA | |
| 153 | **Os-335** | | *Oryza sativa* | Breeding line | NA | |
| 154 | **Os-336** | | *Oryza sativa* | Breeding line | NA | |
| 155 | **Os-337** | | *Oryza sativa* | Variety | NA | |
| 156 | **Os-339** | | *Oryza sativa* | Variety | NA | |
| 157 | **Os-340** | | *Oryza sativa* | Variety | Sothern United States | |
| 158 | **Os-341** | | *Oryza sativa* | Variety | Sothern United States | |
| 159 | **Os-342** | | *Oryza sativa* | Variety | Andra Pradesh, India | |
| 160 | **Os-345** | | *Oryza sativa* | Variety | Punjab, India | |
| 161 | **Os-346** | | *Oryza sativa* | Variety | Sothern United States | |
| 162 | **Os-347** | | *Oryza sativa* | Variety | NA | |
| 163 | **Os-349** | | *Oryza sativa* | Variety | NA | |
| 164 | **Os-351** | | *Oryza sativa* | Land race | Tamil Nadu, India | |
| 165 | **Os-352** | | *Oryza sativa* | Breeding line | NA | |
| 166 | **Os-354** | | *Oryza sativa* | Variety | Sothern United States | |
| 167 | **Os-355** | | *Oryza sativa* | Land race | Tamil Nadu, India | |
| 168 | **Os-357** | | *Oryza sativa* | Breeding line | Wayanad, Kerala, India | |
| 169 | **Os-359** | | *Oryza sativa* | Variety | Sothern United States | |
| 170 | **Os-363** | | *Oryza sativa* | Land race | Tamil Nadu, India | |
| 171 | **Os-371** | | *Oryza sativa* | Breeding line | Sothern United States | |
| 172 | **Os-374** | | *Oryza sativa* | Breeding line | Sothern United States | |
| 173 | **Os-378** | | *Oryza sativa* | Breeding line | Jagtial, ANGRAU | |
| 174 | **Os-381** | | *Oryza sativa* | Breeding line | Rice Research & Development Institute, Batalagoda, Ibbagamuwa*.* | |
| 175 | **Os-382** | | *Oryza sativa* | Breeding line | NA | |
| 176 | **Os-384** | | *Oryza sativa* | Breeding line | NA | |
| 177 | **Os-385** | | *Oryza sativa* | Breeding line | NA | |
| 178 | **Os-387** | | *Oryza sativa* | Variety | Thirupathisaram, TN, India | |
| 179 | **Os-390** | | *Oryza sativa* | Variety | Eastern Himalaya’s, India | |
| 180 | **Os-391** | | *Oryza sativa* | Breeding line | Sri Lanka | |
| 181 | **Os-396** | | *Oryza sativa* | Breeding line | Kerala, India | |
| 182 | **Os-399** | | *Oryza sativa* | Breeding line | NA | |
| 183 | **Os-401** | | *Oryza sativa* | Breeding line | NA | |
| 184 | **Os-412** | | *Oryza sativa* | Land race | Tamil Nadu, India | |
| 185 | **Os-413** | | *Oryza sativa* | Breeding line | NA | |
| 186 | **Os-414** | | *Oryza sativa* | Breeding line | IRRI, Phillipines | |
| 187 | **Os-415** | | *Oryza sativa* | Breeding line | NA | |
| 188 | **Os-416** | | *Oryza sativa* | Breeding line | IRRI, Phillipines | |
| 189 | **Os-417** | | *Oryza sativa* | Variety | NA | |
| 190 | **Os-418** | | *Oryza sativa* | Variety | NA | |
| 191 | **Os-419** | | *Oryza sativa* | Breeding line | IRRI, Phillipines | |
| 192 | **Os-420** | | *Oryza sativa* | Land race | NA | |
| 193 | **Os-421** | | *Oryza sativa* | Breeding line | IRRI, Phillipines | |
| 194 | **Os-423** | | *Oryza sativa* | Breeding line | NA | |
| 195 | **Os-424** | | *Oryza sativa* | Breeding line | NA | |
| 196 | **Os-425** | | *Oryza sativa* | Breeding line | Southern Uninited States | |
| 197 | **Os-426** | | *Oryza sativa* | Breeding line | RARS, Jagtial,(AGRAU),AP | |
| 198 | **Os-428** | | *Oryza sativa* | Breeding line | NA | |
| 199 | **Os-429** | | *Oryza sativa* | Variety | Karnataka, India | |
| 200 | **Os-430** | | *Oryza sativa* | Variety | NA | |
| 201 | **OS-431** | | *Oryza sativa* | Breeding line | NA | |
| 202 | **Os-432** | | *Oryza sativa* | Breeding line | NA | |
| 203 | **Os-433** | | *Oryza sativa* | Breeding line | IRRI, Phillipines | |
| 204 | **Os-435** | | *Oryza sativa* | Breeding line | NA | |
| 205 | **Os-436** | | *Oryza sativa* | Breeding line | NA | |
| 206 | **Os-437** | | *Oryza sativa* | Variety | NA | |
| 207 | **Os-438** | | *Oryza sativa* | Breeding line | NA | |
| 208 | **Os-439** | | *Oryza sativa* | Breeding line | IRRI, Phillipines | |
| 209 | **Os-440** | | *Oryza sativa* | Breeding line | IRRI, Phillipines | |
| 210 | **Os-441** | | *Oryza sativa* | Breeding line | NA | |
| 211 | **Os-442** | | *Oryza sativa* | Breeding line | UP, India | |
| 212 | **OS-443** | | *Oryza sativa* | Breeding line | Northern Thailand | |
| 213 | **Os-445** | | *Oryza sativa* | Breeding line | NA | |
| 214 | **Os-448** | | *Oryza sativa* | Breeding line | NA | |
| 215 | **Os-449** | | *Oryza sativa* | Breeding line | NA | |
| 216 | **Os-450** | | *Oryza sativa* | Breeding line | NA | |
| 217 | **Os-451** | | *Oryza sativa* | Breeding line | NA | |
| 218 | **Os-452** | | *Oryza sativa* | Breeding line | NA | |
| 219 | **Os-453** | | *Oryza sativa* | Variety | NA | |
| 220 | **Os-454** | | *Oryza sativa* | Variety | NA | |
| 221 | **Os-455** | | *Oryza sativa* | Variety | NA | |
| 222 | **Os-456** | | *Oryza sativa* | Breeding line | NA | |
| 223 | **Os-457** | | *Oryza sativa* | Breeding line | Krishidan Seeds Pvt.Ltd, Indore | |
| 224 | **Os-458** | | *Oryza sativa* | Breeding line | Maharastra, India | |
| 225 | **Os-459** | | *Oryza sativa* | Breeding line | IRRI, Phillipines | |
| 226 | **Os-460** | | *Oryza sativa* | Breeding line | TNAU, Coimbatore, TN, India | |
| 227 | **Os-461** | | *Oryza sativa* | Breeding line | IRRI, Phillipines | |
| 228 | **Os-462** | | *Oryza sativa* | Variety | NA | |
| 229 | **Os-463** | | *Oryza sativa* | Variety | IRRI, Phillipines | |
| 230 | **Os-464** | | *Oryza sativa* | Breeding line | CSSRI,Karnal | |
| 231 | **Os-465** | | *Oryza sativa* | Breeding line |  | |
| 232 | **Os-467** | | *Oryza sativa* | Breeding line | Maruteru (ANGRAU) | |
| 233 | **Os-468** | | *Oryza sativa* | Variety | Guntur, AP | |
| 234 | **Os-469** | | *Oryza sativa* | Land race | Southern India | |
| 235 | **Os-470** | | *Oryza sativa* | Variety | NA | |
| 236 | **Os-471** | | *Oryza sativa* | Variety | NA | |
| 237 | **Os-472** | | *Oryza sativa* | Variety | NA | |
| 238 | **Os-473** | | *Oryza sativa* | Variety | Sindh Balochistan, Pakistan | |
| 239 | **Os-474** | | *Oryza sativa* | Variety | RRS, Paiyur, TN, India | |
| 240 | **Os-475** | | *Oryza sativa* | Variety | IRRI, Phillipines | |
| 241 | **Os-476** | | *Oryza sativa* | Breeding line | IRRI, Phillipines | |
| 242 | **Os-477** | | *Oryza sativa* | Variety | NA | |
| 243 | **Os-478** | | *Oryza sativa* | Variety | NA | |
| 244 | **Os-479** | | *Oryza sativa* | Breeding line | Maharastra, India | |
| 245 | **Os-480** | | *Oryza sativa* | Variety | NA | |
| 246 | **Os-481** | | *Oryza sativa* | Variety | NA | |
| 247 | **Os-482** | | *Oryza sativa* | Variety | NA | |
| 248 | **Os-483** | | *Oryza sativa* | Variety | Tamil Nadu, India | |
| 249 | **OS-484** | | *Oryza sativa* | Variety | NA | |
| 250 | **Os-485** | | *Oryza sativa* | Variety | NA | |
| 251 | **Os-486** | | *Oryza sativa* | Variety | IARI, New Delhi, India | |
| 252 | **Os-487** | | *Oryza sativa* | Breeding line | NA | |
| 253 | **Os-489** | | *Oryza sativa* | Breeding line | IRRI, Phillipines | |
| 254 | **Os-490** | | *Oryza sativa* | Landrace | Tamil Nadu, India | |
| 255 | **Os-491** | | *Oryza sativa* | Landrace | Tamil Nadu, India | |
| 256 | **Os-492** | | *Oryza sativa* | Breeding line | Tamil Nadu, India | |
| 257 | **Os-493** | | *Oryza sativa* | Variety | Kerala, India | |
| 258 | **Os-494** | | *Oryza sativa* | Landrace | Tamil Nadu, India | |
| 259 | **Os-495** | | *Oryza sativa* | Landrace | Tamil Nadu, India | |
| 260 | **Os-496** | | *Oryza sativa* | Landrace | Tamil Nadu, India | |
| 261 | **Os-497** | | *Oryza sativa* | Landrace | Tamil Nadu, India | |
| 262 | **Os-498** | | *Oryza sativa* | Landrace | Tamil Nadu, India | |
| 263 | **Os-499** | | *Oryza sativa* | Landrace | Tamil Nadu, India | |
| 264 | **Os-500** | | *Oryza sativa* | Landrace | Tamil Nadu, India | |
| 265 | **Os-501** | | *Oryza sativa* | Landrace | Tamil Nadu, India | |
| 266 | **Os-502** | | *Oryza sativa* | Landrace | Tamil Nadu, India | |
| 267 | **Os-503** | | *Oryza sativa* | Landrace | Tamil Nadu, India | |
| 268 | **Os-505** | | *Oryza sativa* | Landrace | Tamil Nadu, India | |
| 269 | **Os-506** | | *Oryza sativa* | Landrace | Tamil Nadu, India | |
| 270 | **Os-508** | | *Oryza sativa* | Landrace | Tamil Nadu, India | |
| 271 | **Os-509** | | *Oryza sativa* | Landrace | Tamil Nadu, India | |
| 272 | **Os-510** | | *Oryza sativa* | Landrace | Tamil Nadu, India | |
| 273 | **Os-512** | | *Oryza sativa* | Landrace | Tamil Nadu, India | |
| 274 | **Os-513** | | *Oryza sativa* | Landrace | Tamil Nadu, India | |
| 275 | **Os-514** | | *Oryza sativa* | Landrace | Tamil Nadu, India | |
| 276 | **Os-515** | | *Oryza sativa* | Landrace | Tamil Nadu, India | |
| 277 | **Os-516** | | *Oryza sativa* | Landrace | Tamil Nadu, India | |
| 278 | **Os-517** | | *Oryza sativa* | Landrace | Kerala, India | |
| 279 | **Os-518** | | *Oryza sativa* | Landrace | Tamil Nadu, India | |
| 280 | **Os-519** | | *Oryza sativa* | Landrace | Tamil Nadu, India | |
| 281 | **Os-520** | | *Oryza sativa* | Landrace | Tamil Nadu, India | |
| 282 | **Os-521** | | *Oryza sativa* | Landrace | Tamil Nadu, India | |
| 283 | **Os-522** | | *Oryza sativa* | Landrace | Tamil Nadu, India | |
| 284 | **Os-523** | | *Oryza sativa* | Landrace | Tamil Nadu, India | |
| 285 | **Os-524** | | *Oryza sativa* | Landrace | Tamil Nadu, India | |
| 286 | **Os-525** | | *Oryza sativa* | Landrace | Tamil Nadu, India | |
| 287 | **Os-526** | | *Oryza sativa* | Landrace | Tamil Nadu, India | |
| 288 | **Os-527** | | *Oryza sativa* | Landrace | Tamil Nadu, India | |
| 289 | **Os-528** | | *Oryza sativa* | Landrace | Tamil Nadu, India | |
| 290 | **Os-529** | | *Oryza sativa* | Landrace | Tamil Nadu, India | |
| 291 | **Os-530** | | *Oryza sativa* | Landrace | Tamil Nadu, India | |
| 292 | **Os-531** | | *Oryza sativa* | Landrace | Tamil Nadu, India | |
| 293 | **Os-532** | | *Oryza sativa* | Landrace | Tamil Nadu, India | |
| 294 | **Os-533** | | *Oryza sativa* | Landrace | Tamil Nadu, India | |
| 295 | **Os-534** | | *Oryza sativa* | Landrace | Tamil Nadu, India | |
| 296 | **Os-535** | | *Oryza sativa* | Landrace | Tamil Nadu, India | |
| 297 | **Os-536** | | *Oryza sativa* | Landrace | Tamil Nadu, India | |
| 298 | **Os-537** | | *Oryza sativa* | Landrace | Tamil Nadu, India | |
| 299 | **Os-538** | | *Oryza sativa* | Landrace | Tamil Nadu, India | |
| 300 | **Os-539** | | *Oryza sativa* | Landrace | Tamil Nadu, India | |
| 301 | **Os-540** | | *Oryza sativa* | Landrace | Tamil Nadu, India | |
| 302 | **Os-541** | | *Oryza sativa* | Landrace | Tamil Nadu, India | |
| 303 | **Os-542** | | *Oryza sativa* | Landrace | Tamil Nadu, India | |
| 304 | **Os-543** | | *Oryza sativa* | Landrace | Tamil Nadu, India | |
| 305 | **Os-545** | | *Oryza sativa* | Landrace | Tamil Nadu, India | |
| 306 | **Os-546** | | *Oryza sativa* | Landrace | Tamil Nadu, India | |
| 307 | **Os-547** | | *Oryza sativa* | Landrace | Tamil Nadu, India | |
| 308 | **Os-548** | | *Oryza sativa* | Landrace | Tamil Nadu, India | |
| 309 | **Os-549** | | *Oryza sativa* | Landrace | Tamil Nadu, India | |
| 310 | **Os-550** | | *Oryza sativa* | Landrace | Tamil Nadu, India | |
| 311 | **Os-551** | | *Oryza sativa* | Landrace | Tamil Nadu, India | |
| 312 | **Os-552** | | *Oryza sativa* | Landrace | Tamil Nadu, India | |
| 313 | **Os-553** | | *Oryza sativa* | Landrace | Tamil Nadu, India | |
| 314 | **Os-554** | | *Oryza sativa* | Landrace | Tamil Nadu, India | |
| 315 | **Os-555** | | *Oryza sativa* | Landrace | Tamil Nadu, India | |
| 316 | **Os-556** | | *Oryza sativa* | Landrace | Tamil Nadu, India | |
| 317 | **Os-557** | | *Oryza sativa* | Landrace | West Bengal, India | |
| 318 | **Os-558** | | *Oryza sativa* | Landrace | West Bengal, India | |
| 319 | **Os-560** | | *Oryza sativa* | Landrace | Tamil Nadu, India | |
| 320 | **Os-561** | | *Oryza sativa* | Landrace | Tamil Nadu, India | |
| 321 | **Os-562** | | *Oryza sativa* | Landrace | West Bengal, India | |
| 322 | **Os-563** | | *Oryza sativa* | Landrace | Tamil Nadu, India | |
| 323 | **Os-564** | | *Oryza sativa* | Landrace | Tamil Nadu, India | |
| 324 | **Os-565** | | *Oryza sativa* | Landrace | West Bengal, India | |
| 325 | **Os-566** | | *Oryza sativa* | Landrace | Tamil Nadu, India | |
| 326 | **Os-567** | | *Oryza sativa* | Variety | West Bengal, India | |
| 327 | **Os-568** | | *Oryza sativa* | Variety | Tamil Nadu, India | |
| 328 | **Os-569** | | *Oryza sativa* | Landrace | West Bengal, India | |
| 329 | **Os-570** | | *Oryza sativa* | Variety | Tamil Nadu, India | |
| 330 | **Os-571** | | *Oryza sativa* | Landrace | Tamil Nadu, India | |
| 331 | **Os-572** | | *Oryza sativa* | Landrace | Tamil Nadu, India | |
| 332 | **Os-573** | | *Oryza sativa* | Variety | NA | |
| 333 | **Os-574** | | *Oryza sativa* | Landrace | Tamil Nadu, India | |
| 334 | **Os-575** | | *Oryza sativa* | Landrace | Tamil Nadu, India | |
| 335 | **Os-576** | | *Oryza sativa* | Variety | NA | |
| 336 | **Os-577** | | *Oryza sativa* | Variety | Jammu and Kashmir, India | |
| 337 | **Os-578** | | *Oryza sativa* | Land races | Tamil Nadu, India | |
| 338 | **Os-579** | | *Oryza sativa* | Landrace | Orissa, India | |
| 339 | **Os-580** | | *Oryza sativa* | Landrace | Tamil Nadu, India | |
| 340 | **Os-582** | | *Oryza sativa* | Landrace | Tamil Nadu, India | |
| 341 | **Os-583** | | *Oryza sativa* | Variety | Kerala, India | |
| 342 | **Os-584** | | *Oryza sativa* | Variety | UAS, Bangalore, India | |
| 343 | **Os-585** | | *Oryza sativa* | Breeding line | IRRI, Philippines | |
| 344 | **Os-586** | | *Oryza sativa* | Breeding line | IRRI, Philippines | |
| 345 | **Os-588** | | *Oryza sativa* | Variety | Haryana, India | |
| 346 | **Os-589** | | *Oryza sativa* | Variety | Uttarkhand, India | |
| 347 | **Os-590** | | *Oryza sativa* | Land race | Tamil Nadu, India | |
| 348 | **Os-591** | | *Oryza sativa* | Variety | Orissa, India | |
| 349 | **Os-592** | | *Oryza sativa* | Land race | Tamil Nadu, India | |
| 350 | **Os-593** | | *Oryza sativa* | Land race | Tamil Nadu, India | |
| 351 | **Os-594** | | *Oryza sativa* | Land race | IRRI, Philippines | |
| 352 | **Os-595** | | *Oryza sativa* | Land race | Tamil Nadu, India | |
| 353 | **Os-597** | | *Oryza sativa* | Variety | Brazil | |
| 354 | **Os-599** | | *Oryza sativa* | Variety | Manipur | |
| 355 | **Os-600** | | *Oryza sativa* | Breeding line | IRRI, Philippines | |
| 356 | **Os-601** | | *Oryza sativa* | Variety | Uttarkhand, India | |
| 357 | **Os-603** | | *Oryza sativa* | Breeding line | IRRI, Philippines | |
| 358 | **Os-604** | | *Oryza sativa* | Landrace | Tamil Nadu, India | |
| 359 | **Os-608** | | *Oryza sativa* | Variety | UAS, Bangalore, India | |
| 360 | **Os-609** | | *Oryza sativa* | Breeding line | IRRI, Philippines | |
| 361 | **Os-610** | | *Oryza sativa* | Variety | West Bengal, India | |
| 362 | **Os-611** | | *Oryza sativa* | Breeding line | IRRI, Philippines | |
| 363 | **Os-613** | | *Oryza sativa* | Land race | Tamil Nadu, India | |
| 364 | **Os-614** | | *Oryza sativa* | Variety | Uttarkhand, India | |
| 365 | **Os-617** | | *Oryza sativa* | Variety | Uttarkhand, India | |
| 366 | **Os-618** | | *Oryza sativa* | Variety | Kerala, India | |
| 367 | **Os-623** | | *Oryza sativa* | Breeding line | Taiwan | |
| 368 | **Os-625** | | *Oryza sativa* | Land race | Tamil Nadu, India | |
| 369 | **Os-627** | | *Oryza sativa* | Breeding line | IRRI, Philippines | |
| 370 | **Os-628** | | *Oryza sativa* | Breeding line | Venezuela | |
| 371 | **Os-630** | | *Oryza sativa* | Breeding line | IRRI, Philippines | |
| 372 | **Os-631** | | *Oryza sativa* | Land races | NA | |
| 373 | **Os-632** | | *Oryza sativa* | Variety | Andhra Pradesh | |
| 374 | **Os-634** | | *Oryza sativa* | Land race | Tamil Nadu, India | |
| 375 | **Os-635** | | *Oryza sativa* | Breeding line | IRRI, Philippines | |
| 376 | **Os-636** | | *Oryza sativa* | Breeding line | Colombia | |
| 377 | **Os-637** | | *Oryza sativa* | Breeding line | IRRI, Philippines | |
| 378 | **Os-638** | | *Oryza sativa* | Variety | Orissa, India | |
| 379 | **Os-640** | | *Oryza sativa* | Breeding line | Uruguay | |
| 380 | **Os-641** | | *Oryza sativa* | Breeding line | IRRI, Philippines | |
| 381 | **Os-643** | | *Oryza sativa* | Breeding line | Tamil Nadu, India | |
| 382 | **Os-644** | | *Oryza sativa* | Land race | Tamil Nadu, India | |
| 383 | **Os-645** | | *Oryza sativa* | Landrace | Tamil Nadu, India | |
| 384 | **Os-646** | | *Oryza sativa* | Breeding line | IRRI, Philippines | |
| 385 | **Os-647** | | *Oryza sativa* | Breeding line | Cote D’Ivoire | |
| 386 | **Os-648** | | *Oryza sativa* | Breeding line | Puerto Rico | |
| 387 | **Os-650** | | *Oryza sativa* | Variety | Bangladesh | |
| 388 | **Os-651** | | *Oryza sativa* | Breeding line | IRRI, Philippines | |
| 389 | **Os-652** | | *Oryza sativa* | Variety | Haryana, India | |
| 390 | **Os-653** | | *Oryza sativa* | Breeding line | IRRI, Philippines | |
| 391 | **Os-654** | | *Oryza sativa* | Breeding line | IRRI, Philippines | |
| 392 | **Os-655** | | *Oryza sativa* | Land race | Tamil Nadu, India | |
| 393 | **Os-656** | | *Oryza sativa* | Breeding line | IRRI, Philippines | |
| 394 | **Os-657** | | *Oryza sativa* | Breeding line | Nigeria | |
| 395 | **Os-658** | | *Oryza sativa* | Variety | Orissa, India | |
| 396 | **Os-659** | | *Oryza sativa* | Land race | Tamil Nadu, India | |
| 397 | **Os-660** | | *Oryza sativa* | Breeding line | IRRI, Philippines | |
| 398 | **Os-661** | | *Oryza sativa* | Breeding line | IRRI, Philippines | |
| 399 | **Os-662** | | *Oryza sativa* | Breeding line | United States | |
| 400 | **Os-663** | | *Oryza sativa* | Breeding line | Honduras | |
| 401 | **Os-664** | | *Oryza sativa* | Breeding line | Andhra Pradesh | |
| 402 | **Os-665** | | *Oryza sativa* | Breeding line | Jamaica | |
| 403 | **Os-667** | | *Oryza sativa* | Breeding line | Srilanka | |
| 404 | **Os-669** | | *Oryza sativa* | Breeding line | IRRI, Philippines | |
| 405 | **Os-670** | | *Oryza sativa* | Breeding line | IRRI, Philippines | |
| 406 | **Os-671** | | *Oryza sativa* | Breeding line | Argentina | |
| 407 | **Os-672** | | *Oryza sativa* | Land race | Tamil Nadu, India | |
| 408 | **Os-673** | | *Oryza sativa* | Variety | Kerala, India | |
| 409 | **Os-674** | | *Oryza sativa* | Breeding line | United States | |
| 410 | **Os-675** | | *Oryza sativa* | Variety | West Bengal, India | |
| 411 | **Os-676** | | *Oryza sativa* | Breeding line | India | |
| 412 | **Os-677** | | *Oryza sativa* | Breeding line | Belize | |
| 413 | **Os-678** | | *Oryza sativa* | Breeding line | IRRI, Philippines | |
| 414 | **Os-679** | | *Oryza sativa* | Breeding line | United States | |
| 415 | **Os-680** | | *Oryza sativa* | Land race | Tamil Nadu, India | |
| 416 | **Os-681** | | *Oryza sativa* | Breeding line | IRRI, Philippines | |
| 417 | **Os-682** | | *Oryza sativa* | Land race | Tamil Nadu, India | |
| 418 | **Os-683** | | *Oryza sativa* | Variety | TRRI, Aduthurai, TN, India | |
| 419 | **Os-684** | | *Oryza sativa* | Variety | UAS, Bangalore, India | |
| 420 | **Os-685** | | *Oryza sativa* | Variety | Haryana, | |
| 421 | **Os-686** | | *Oryza sativa* | Breeding line | IRRI, Philippines | |
| 422 | **Os-687** | | *Oryza sativa* | Land race | Orissa, India | |
| 423 | **Os-689** | | *Oryza sativa* | Breeding line | Bangladesh | |
| 424 | **Os-690** | | *Oryza sativa* | Variety | Jammu and Kashmir, India | |
| 425 | **Os-691** | | *Oryza sativa* | Variety | Karnataka, India | |
| 426 | **Os-692** | | *Oryza sativa* | Breeding line | IRRI, Philippines | |
| 427 | **Os-693** | | *Oryza sativa* | Breeding line | IRRI, Philippines | |
| 428 | **Os-694** | | *Oryza sativa* | Breeding line | Indonesia | |
| 429 | **Os-695** | | *Oryza sativa* | Land race | Tamil Nadu, India | |
| 430 | **Os-696** | | *Oryza sativa* | Breeding line | Indonesia | |
| 431 | **Os-697** | | *Oryza sativa* | Breeding line | Bulgaria | |
| 432 | **Os-698** | | *Oryza sativa* | Breeding line | Brazil | |
| 433 | **Os-699** | | *Oryza sativa* | Variety | UAS, Bangalore, India | |
| 434 | **Os-700** | | *Oryza sativa* | Variety | West Bengal, India | |
| 435 | **Os-701** | | *Oryza sativa* | Breeding line | Indonesia | |
| 436 | **Os-703** | | *Oryza sativa* | Variety | Tamil Nadu, India | |
| 437 | **Os-705** | | *Oryza sativa* | Breeding line | Indonesia | |
| 438 | **Os-706** | | *Oryza sativa* | Land race | Tamil Nadu, India | |
| 439 | **Os-707** | | *Oryza sativa* | Variety | Andhra Pradesh, India | |
| 440 | **Os-708** | | *Oryza sativa* | Breeding line | Indonesia | |
| 441 | **Os-710** | | *Oryza sativa* | Breeding line | IRRI, Philippines | |
| 442 | **Os-713** | | *Oryza sativa* | Breeding line | China | |
| 443 | **Os-715** | | *Oryza sativa* | Land race | Tamil Nadu, India | |
| 444 | **Os-717** | | *Oryza sativa* | Land race | Tamil Nadu, India | |
| 445 | **Os-718** | | *Oryza sativa* | Land race | Tamil Nadu, India | |
| 446 | **Os-719** | | *Oryza sativa* | Land race | Tamil Nadu, India | |
| 447 | **Os-720** | | *Oryza sativa* | Land race | Tamil Nadu, India | |
| 448 | **Os-721** | | *Oryza sativa* | Breeding line | TRRI, Aduthurai, TN, India | |
| 449 | **Os-728** | | *Oryza sativa* | Breeding line | TRRI, Aduthurai, TN, India | |
| 450 | **Os-729** | | *Oryza sativa* | Variety | Tamil Nadu, India | |
| 451 | **Os-732** | | *Oryza sativa* | Land race | Tamil Nadu, India | |
| 452 | **Os-733** | | *Oryza sativa* | Breeding line | TRRI, Aduthurai, TN, India | |
| 453 | **Os-734** | | *Oryza sativa* | Land race | Tamil Nadu, India | |
| 454 | **Os-737** | | *Oryza sativa* | Breeding line | IRRI, Philippines | |
| 455 | **Os-739** | | *Oryza sativa* | Breeding line | TRRI, Aduthurai, TN, India | |
| 456 | **Os-740** | | *Oryza sativa* | Breeding line | TRRI, Aduthurai, TN, India | |
| 457 | **Os-741** | | *Oryza sativa* | Breeding line | TRRI, Aduthurai, TN, India | |
| 458 | **Os-743** | | *Oryza sativa* | Breeding line | IRRI, Philippines | |
| 459 | **Os-745** | | *Oryza sativa* | Land race | Tamil Nadu, India | |
| 460 | **Os-746** | | *Oryza sativa* | Land race | Tamil Nadu, India | |
| 461 | **Os-747** | | *Oryza sativa* | Land race | Tamil Nadu, India | |
| 462 | **Os-748** | | *Oryza sativa* | Land race | Tamil Nadu, India | |
| 463 | **Os-749** | | *Oryza sativa* | Variety | IRRI, Philippines | |
| 464 | **Os-753** | | *Oryza sativa* | Breeding line | IRRI, Philippines | |
| 465 | **Os-758** | | *Oryza sativa* | Breeding line | IRRI, Philippines | |
| 466 | **Os-760** | | *Oryza sativa* | Breeding line | IRRI, Philippines | |
| 467 | **Os-762** | | *Oryza sativa* | Breeding line | TRRI, Aduthurai, TN, India | |
| 468 | **Os-763** | | *Oryza sativa* | Breeding line | IRRI, Philippines | |
| 469 | **Os-764** | | *Oryza sativa* | Land race | IRRI, Philippines | |
| 470 | **Os-765** | | *Oryza sativa* | Land race | Tamil Nadu, India | |
| 471 | **Os-766** | | *Oryza sativa* | Land race | Tamil Nadu, India | |
| 472 | **Os-768** | | *Oryza sativa* | Land race | Tamil Nadu, India | |
| 473 | **Os-769** | | *Oryza sativa* | Land race | Tamil Nadu, India | |
| 474 | **Os-770** | | *Oryza sativa* | Land race | Tamil Nadu, India | |
| 475 | **Os-771** | | *Oryza sativa* | Breeding line | TRRI, Aduthurai, TN, India | |
| 476 | **Os-772** | | *Oryza sativa* | Land race | Tamil Nadu, India | |
| 477 | **Os-773** | | *Oryza sativa* | Land race | Tamil Nadu, India | |
| 478 | **Os-774** | | *Oryza sativa* | Land race | Tamil Nadu, India | |
| 479 | **Os-775** | | *Oryza sativa* | Land race | Tamil Nadu, India | |
| 480 | **Os-776** | | *Oryza sativa* | Land race | Kerala, India | |
| 481 | **Os-778** | | *Oryza sativa* | Breeding line | Bangladesh | |
| 482 | **Os-779** | | *Oryza sativa* | Breeding line | Tamil Nadu, India | |
| 483 | **Os-780** | | *Oryza sativa* | Breeding line | Tamil Nadu, India | |
| 484 | **Os-781** | | *Oryza sativa* | Land race | Tamil Nadu, India | |
| 485 | **Os-782** | | *Oryza sativa* | Land race | Tamil Nadu, India | |
| 486 | **Os-783** | | *Oryza sativa* | Land race | Tamil Nadu, India | |
| 487 | **Os-784** | | *Oryza sativa* | Breeding line | Tamil Nadu, India | |
| 488 | **Os-785** | | *Oryza sativa* | Land race | Tamil Nadu, India | |
| 489 | **Os-787** | | *Oryza sativa* | Land race | Tamil Nadu, India | |
| 490 | **Os-789** | | *Oryza sativa* | Breeding line | Tamil Nadu, India | |
| 491 | **Os-790** | | *Oryza sativa* | Land race | Tamil Nadu, India | |
| 492 | **Os-791** | | *Oryza sativa* | Land race | Tamil Nadu, India | |
| 493 | **Os-792** | | *Oryza sativa* | Breeding line | IRRI, Philippines | |
| 494 | **Os-793** | | *Oryza sativa* | Land race | Tamil Nadu, India | |
| 495 | **Os-794** | | *Oryza sativa* | Land race | Tamil Nadu, India | |
| 496 | **Os-795** | | *Oryza sativa* | Breeding line | IRRI, Philippines | |
| 497 | **Os-796** | | *Oryza sativa* | Land race | Tamil Nadu, India | |
| 498 | **Os-797** | | *Oryza sativa* | Land race | Tamil Nadu, India | |
| 499 | **Os-798** | | *Oryza sativa* | Breeding line | IRRI, Philippines | |
| 500 | **Os-799** | | *Oryza sativa* | Land race | Tamil Nadu, India | |
| 501 | **Os-800** | | *Oryza sativa* | Breeding line | IRRI, Philippines | |
| 502 | **Os-801** | | *Oryza sativa* | Breeding line | Tamil Nadu, India | |
| 503 | **Os-802** | | *Oryza sativa* | Breeding line | Tamil Nadu, India | |
| 504 | **Os-812** | | *Oryza sativa* | Breeding line | IRRI, Philippines | |
| 505 | **Os-819** | | *Oryza sativa* | Breeding line | United States | |
| 506 | **Os-821** | | *Oryza sativa* | Variety | Haryana, India | |
| 507 | **Os-822** | | *Oryza sativa* | Land race | Tamil Nadu, India | |
| 508 | **Os-823** | | *Oryza sativa* | Breeding line | IRRI, Philippines | |
| 509 | **Os-824** | | *Oryza sativa* | Variety | NA | |
| 510 | **Os-825** | | *Oryza sativa* | Breeding line | IRRI, Philippines | |
| 511 | **Os-826** | | *Oryza sativa* | Land race | Tamil Nadu, India | |
| 512 | **Os-827** | | *Oryza sativa* | Breeding line | IRRI, Philippines | |
|  | Internal control | | | | | |
| 513 | **RSM 271** | *Oryza sativa* | | Mutant line | | TNAU, Tamil Nadu, India |
| 514 | **RSM 311** | *Oryza sativa* | | Mutant line | | TNAU, Tamil Nadu, India |
| 515 | **Pooja** | *Oryza sativa* | | Variety | | Andhra Pradesh, India |

NA- information not available
