# Supplementary material for: EcoTILLING by sequencing reveals polymorphisms in genes encoding starch synthases that are associated with low glycemic response in rice
Source: BMC Plant Biol. 2017 Jan 14;17:13. doi: 10.1186/s12870-016-0968-0 (PMC5423428; doi:10.1186/s12870-016-0968-0)
Supplement: Supplementary file 2 — PCR reaction conditions followed for the amplification of EcoTILLING fragments. (DOCX 14 kb) [file 12870_2016_968_MOESM2_ESM.docx]

**Table S4. PCR reaction conditions followed for the amplification of EcoTILLING fragments**

| **S.No** | **Reaction Step** | **Temperature (°C)** | **Time** | **Cycles** |
| --- | --- | --- | --- | --- |
| 1. | Initial denaturation | 94 | 30 seconds | 1 |
| 2. | Denaturation | 94 | 30 seconds | 10 |
| 3. | Annealing with 0.6°C  decrement per cycle | 65^*^/62^**^ | 50 seconds |  |
| 4. | Extension | 65 | 1min/kb (Fixed based on EcoTILLING fragment size) |  |
| 5. | Denaturation | 94 | 30 sec | 30 |
| 6. | Annealing | 57 | 1minutes |  |
| 7. | Extension | 65 | 1min/kb |  |
| 8. | Final extension | 65 | 10 minutes | 1 |
| 9. | Hold at | 10 | Till end |  |

^*^PCR cycle I

^**^PCR cycle II
