# Supplementary material for: EcoTILLING by sequencing reveals polymorphisms in genes encoding starch synthases that are associated with low glycemic response in rice
Source: BMC Plant Biol. 2017 Jan 14;17:13. doi: 10.1186/s12870-016-0968-0 (PMC5423428; doi:10.1186/s12870-016-0968-0)
Supplement: Supplementary file 3 — PCR cycle condition and composition standardized for the amplification of different EcoTILLING fragments in candidate genes. (DOCX 14 kb) [file 12870_2016_968_MOESM3_ESM.docx]

**Table S5. PCR cycle condition and composition standardized for the amplification of different EcoTILLING fragments in candidate genes**

| **S.No** | **Candidate gene/ EcoTILLING fragment** | **GC content (%)** | **PCR** | |
| --- | --- | --- | --- | --- |
|  |  |  | **Cycle Condition** | **Composition of PCR master mix** |
| 1. | *GBSS I* | 48.27 | PCR Cycle I | PCR master mix composition I |
| 2. | *SS I* | 41.61 | PCR cycle II | PCR master mix composition I |
| 3. | *SS IIa* | | | |
|  | EcoTILLING fragment I | 62.78 | PCR Cycle I | PCR master mix composition II |
|  | EcoTILLING fragment II | 53.55 | PCR cycle II | PCR master mix composition I |
| 4. | *SS IIIa* |  |  |  |
|  | Eco-TILLING Fragment 1 | 40.39 | PCR cycle II | PCR master mix composition II |
|  | EcoTILLING Fragment 2 | 45.74 | PCR cycle II | PCR master mix composition II |
| 5. | *SBE Ia* | 39.94 | PCR Cycle I | PCR master mix composition II |
| 6. | *SBE IIb* | | | |
|  | EcoTILLING Fragment 1 | 37.60 | PCR cycle II | PCR master mix composition II |
|  | EcoTILLING Fragment 2 | 35.27 | PCR cycle II | PCR master mix composition II |
