# Supplementary material for: EcoTILLING by sequencing reveals polymorphisms in genes encoding starch synthases that are associated with low glycemic response in rice
Source: BMC Plant Biol. 2017 Jan 14;17:13. doi: 10.1186/s12870-016-0968-0 (PMC5423428; doi:10.1186/s12870-016-0968-0)
Supplement: Supplementary file 4 — Super pool wise sequencing statistics. (DOCX 15 kb) [file 12870_2016_968_MOESM4_ESM.docx]

**Table S6. Super pool wise sequencing statistics**

| **S.No** | **Super pool ID** | **No. of reads (in millions)** | **No. of bases in millions with quality score ≥ 20** | **Average length of reads (bp)** | **Coverage per accession** |
| --- | --- | --- | --- | --- | --- |
| 1. | C1 | 5.30 | 367.2 | 91 | 282.5 |
| 2. | C2 | 4.59 | 335.8 | 97 | 257.9 |
| 3. | C3 | 2.20 | 161.8 | 98 | 123.9 |
| 4. | C4 | 4.54 | 317.0 | 93 | 244.0 |
| 5. | C5 | 4.33 | 294.6 | 91 | 226.3 |
| 6. | C6 | 4.15 | 255.2 | 85 | 196.3 |
| 7. | C7 | 5.38 | 337.7 | 81 | 259.4 |
| 8. | C8 | 5.48 | 472.9 | 88 | 363.4 |
| 9. | R1 | 6.00 | 405.4 | 89 | 311.8 |
| 10. | R2 | 5.25 | 349.2 | 90 | 268.7 |
| 11. | R3 | 4.14 | 259.2 | 88 | 199.4 |
| 12. | R4 | 4.62 | 358.7 | 92 | 275.6 |
| 13. | R5 | 6.82 | 477.4 | 96 | 367.22 |
| 14. | R6 | 6.96 | 478.6 | 90 | 368.0 |
| 15. | R7 | 2.82 | 224.3 | 88 | 172.4 |
| 16. | R8 | 5.71 | 401.2 | 90 | 308.7 |
| Mean | | 4.89 | 343.5 | 90.4 | 264.09 |
