# Supplementary material for: EcoTILLING by sequencing reveals polymorphisms in genes encoding starch synthases that are associated with low glycemic response in rice
Source: BMC Plant Biol. 2017 Jan 14;17:13. doi: 10.1186/s12870-016-0968-0 (PMC5423428; doi:10.1186/s12870-016-0968-0)
Supplement: Supplementary file 5 — Gene wise list of sequence variants discovered through EcoTILLING by sequencing. (DOC 107 kb) [file 12870_2016_968_MOESM5_ESM.doc]

**Table S7**. Gene wise list of sequence variants discovered through EcoTILLING by sequencing

| **S.No** | **Gene Name** | **Nucleotide position change** | **Reference Base** | **Called Base** | **Type of variant** | **Position of variant** | **Germplasm accessions deconvolved through Sanger sequencing** |
| --- | --- | --- | --- | --- | --- | --- | --- |
|  | ***GBSS I* EcoTILLING fragment** | | | | | | |
| 1. | *GBSS1* | 1019 | C | - | Indel | Intron | Os-793 |
| 2. | *GBSS1* | 1097 | C | T | SNP | Intron | Os-032 |
| 3. | *GBSS1* | 1804 | T | C | SNP | Exon | Os-599 |
| 4. | *GBSSI* | 2221 | G | A | SNP | Intron | Os-111, Os-173 |
|  | ***SS I* EcoTILLING fragment** | | | | | | |
| 5. | *SSI* | 328 | C | - | Indel | Intron | Os-531 |
| 6. | *SSI* | 388 | T | C | SNP | Intron | OS-223 |
| 7. | *SSI* | 444 | A | G | SNP | Intron | Os-381 |
| 8. | *SSI* | 507 | T | G | SNP | Intron | Os-144 |
| 9. | *SSI* | 571 | T | C | SNP | Intron | Os-802 |
| 10. | *SSI* | 620 | G | A | SNP | Intron | Os-110 |
| 11. | *SSI* | 717 | A | G | SNP | Intron | Os-448, Os-429 |
| 12. | *SSI* | 731 | T | C | SNP | Intron | Os-202 |
| 13. | *SSI* | 832 | G | C | SNP | Intron | Os-658 |
| .14. | *SSI* | 885 | T | G | SNP | Intron | Os-010 |
| .15. | *SSI* | 927 | G | - | Indel | Intron | Os-799 |
| 16. | *SSI* | 979 | A | - | Indel | Intron | Os-101 |
| 17. | *SSI* | 980 | C | |- | Indel | Intron | Os-503, Os-454 |
| 18. | *SSI* | 981 | C | |- | Indel | Intron | Os-523 |
| 19. | *SSI* | 1023 | A | G | SNP | Intron | Os-101 |
| 20. | *SSI* | 1116 | G | A | SNP | Intron | Os-390 |
| 21. | *SSI* | 1449 | T | C | SNP | Exon | Os-441 |
| 22. | *SSI* | 1620 | T | C | SNP | Intron | Os-018 |
| 23. | *SSI* | 1664 | T | C | SNP | Intron | Os-382 |
| 24. | *SSI* | 1737 | A | G | SNP | Intron | Os-098 |
| 25. | *SSI* | 1836 | C | A | SNP | Intron | Os-506 |
| 26. | *SSI* | 1903 | G | A | SNP | Intron | Os-821 |
| 27. | *SSI* | 1980 | G | A | SNP | Intron | Os-331 |
| 28. | *SSI* | 2180 | G | C | SNP | Intron | Os-413, Os-152 |
| 29. | *SSI* | 2198 | A | G | SNP | Intron | Os-535 |
| 30. | *SSI* | 2252 | T | C | SNP | Exon | Os-008 |
| 31. | *SSI* | 2288 | C | A | SNP | Exon | Os-048 |
| 32. | *SSI* | 2371 | C | T | SNP | Intron | Os-426 |
| 33. | *SSI* | 2432 | T | A | SNP | Exon | Os-107 |
| 34. | *SSI* | 2573 | A | - | Indel | Intron | Os-339 |
| 35. | *SSI* | 2584 | C | A | SNP | Intron | Os-415, Os-480 |
| 36. | *SSI* | 2651 | G | A | SNP | Intron | Os-129 |
| 37. | *SSI* | 2726 | T | G | SNP | Intron | Os-662 |
| 38. | *SSI* | 2947 | G | A | SNP | Intron | Os-340 |
| 39. | *SSI* | 2996 | C | T | SNP | Intron | Os-069, Os-536 |
| 40. | *SSI* | 3072 | G | A | SNP | Intron | Os-563 |
| 41. | *SSI* | 3138 | C | T | SNP | Intron | Os-799 |
| 42. | *SSI* | 3160 | T | A | SNP | Intron | Os-588 |
| 43. | *SSI* | 3224 | A | G | SNP | Intron | Os-823 |
| **44.** | ***SSI*** | **3538** | **G** | **A** | **SNP** | **Exon** | **Os-578, Os-631, RSM 311** |
| 45. | *SSI* | 3785 | T | - | Indel | Intron | Os-105 |
| 46. | *SSI* | 3786 | T | |- | Indel | Intron | Os-092 |
| 47. | *SSI* | 3937 | G | A | SNP | Exon | Os-425 |
| 48. | *SSI* | 4015 | T | G | SNP | Intron | Os-782 |
| **49.** | ***SSI*** | **4127** | **T** | **C** | **SNP** | **Exon** | **Os-468, Os-678, Os-076** |
|  | ***SSIIa* EcoTILLING fragment-1^st^amplicon** | | | | | | |
| 50. | *SSIIa* | 516 | T | C | SNP | Exon | Os-178 |
| 51. | *SSIIa* | 776 | A | G | SNP | Intron | Os-086, |
| 52. | *SSIIa* | 851 | C | A | SNP | Intron | Os-429 |
| 53. | *SSIIa* | 1049 | A | - | Indel | Intron | Os-229 |
|  | ***SSIIa*EcoTILLING fragment-2^nd^amplicon** | | | | | | |
| **54.** | ***SSIIa*** | **3761** | **G** | **-** | **Indel** | **Exon** | **Os-351** |
| **55.** | ***SSIIa*** | **3797** | **G** | **A** | **SNP** | **Exon** | **Os-468, Os-211, RSM 271** |
| 56. | *SSIIa* | 3901 | T | G | SNP | Exon | Os-433 |
| **57.** | ***SSIIa*** | **4196** | **G** | **A** | **SNP** | **Exon** | **Os-363, Os-495** |
|  | ***SSIIIa* EcoTILLING fragment-1^st^amplicon** | | | | | | |
| 58. | *SSIIIa* | 2276 | T | C | SNP | Exon | Os-058 |
| 59. | *SSIIIa* | 3135 | C | C|A | SNP | Exon | Os-100 |
| **60.** | ***SSIIIa*** | **3559** | **T** | **A** | **SNP** | **Exon** | **OS-578, Os-495, Os-468, RSM 271, RSM 311** |
|  | ***SSIIIa* EcoTILLING fragment-2^nd^amplicon** | | | | | | |
| 61. | *SSIIIa* | 9076 | C | A | SNP | Intron | Os-068 |
| 62. | *SSIIIa* | 9467 | T | C | SNP | Intron | Os-819 |
| 63. | *SSIIIa* | 9517 | T | C | SNP | Intron | Os-586 |
| 64. | *SSIIIa* | 10336 | G | A | SNP | Intron | Os-050 |
|  | **SBE Ia EcoTILLING fragment** | | | | | | |
| 65. | *SBEIa* | 1137 | C | T | SNP | Intron | Os-029 |
| 66. | *SBEIa* | 1569 | C | A | SNP | Exon | Os-717 |
| 67. | *SBEIa* | 2337 | C | T | SNP | Intron | Os-225 |
|  | ***SBEIIb* EcoTILLING fragment-1^st^amplicon** | | | | | | |
| 68. | *SBEIIb* | 1950 | C | - | Indel | Intron | Os-387 |
| 69. | *SBEIIb* | 2648 | T | G | SNP | Intron | Os-336 |
| 70. | *SBEIIb* | 2857 | T | G | SNP | Intron | Os-012, Os-092 |
|  | ***SBEIIb* EcoTILLING fragment-2^nd^amplicon** | | | | | | |
| 71. | *SBEIIb* | 9536 | A | G | SNP | Intron | Os-056, |
| 72. | *SBEIIb* | 10363 | A | C | SNP | Intron | Os-035 |
