# Supplementary material for: EcoTILLING by sequencing reveals polymorphisms in genes encoding starch synthases that are associated with low glycemic response in rice
Source: BMC Plant Biol. 2017 Jan 14;17:13. doi: 10.1186/s12870-016-0968-0 (PMC5423428; doi:10.1186/s12870-016-0968-0)
Supplement: Supplementary file 6 — Details of gene specific primers designed to amplify EcoTILLING fragments. (DOCX 14 kb) [file 12870_2016_968_MOESM6_ESM.docx]

**Table S2. Details of gene specific primers designed to amplify EcoTILLING fragments**

| **Gene** | **Primers (5’ to 3’)** | **Amplicon size (Kb)** |
| --- | --- | --- |
| *GBSS I* | F – TTGCAGACAGGTACGAGAGG | 1.910 |
|  | R– CTGCAGCTGGATGAGTCCAC |  |
| *SS I* | F – GGGGATGGGGATCGGGG | 4.138 |
|  | R – CCGGACAGGTCATCAACAGA |  |
| *SS IIa* | F_1_– GTCTTGAAGCGGAGGGGC | 0.661 |
|  | R_1_– AGGGAGAACATTCAGCAGCC |  |
|  | F_2_ – GGTGTGCCCTATGGGGATG | 1.540 |
|  | R_2_ – TCACCATTGGTACTTGGCCTT |  |
| *SS IIIa* | F_1_ – AGCTGGGGCAGAAGATGATG | 2.718 |
|  | R_1_ – GTGCTAGGCTCTATGTGCCA |  |
|  | F_2_ – ACTGCCATGATTGGTCAAGTG | 1.551 |
|  | R_2_– CAGGGCGTAATCCACACCAT |  |
| *SBE Ia* | F– AGTTTGGGATTAATACAGTTGATGG | 2.411 |
|  | R – TAAGTAGCCATCACCTCCAAGG |  |
| *SBE IIb* | F_1_ – CAAGTGACGTTGAAGGCGTG | 2.226 |
|  | R_1_– TATGGGATTTCTCCTGCGGC |  |
|  | F_2_ – GTGGAATGCCTACATTTGCCC | 3.240 |
|  | R_2_ – GTGAAGTGCTCTGCAGTGTG |  |

F= Forward primers

R= Reverse primers
