# Supplementary material for: EcoTILLING by sequencing reveals polymorphisms in genes encoding starch synthases that are associated with low glycemic response in rice
Source: BMC Plant Biol. 2017 Jan 14;17:13. doi: 10.1186/s12870-016-0968-0 (PMC5423428; doi:10.1186/s12870-016-0968-0)
Supplement: Supplementary file 7 — PCR compositions followed for the amplification of EcoTILLING fragments. (DOCX 14 kb) [file 12870_2016_968_MOESM7_ESM.docx]

**Table S3. PCR compositions followed for the amplification of EcoTILLING fragments**

| **S.No** | **Reagent** | **Concentration** | **Volume (µl)** |
| --- | --- | --- | --- |
| 1. | LongAMPTaq Reaction buffer | 5x | 10 |
| 2. | LongAmp®Taq polymerase | 2.5 units | 2^*^/2.5^**^ |
| 3. | dNTPs | 10mM | 1.5 |
| 4. | DMSO | - | 2 |
| 5. | Mgcl_2_ | 50mM | 2^*^/3^**^ |
| 6. | Template DNA | 50 ng/µl | 5^*^/7^**^ |
| 7. | Forward EcoTILLING primer | 10 mM | 2 |
| 8. | Reverse EcoTILLING primer | 10 mM | 2 |
| 9. | H_2_O | - | 23.5^*^/20.0^**^ |
| 10. | Total reaction volume | | 50.00 |

^*^PCR master mix composition I

^**^PCR master mix composition II
